# Enhanced Energy Metabolism and Developmental Signaling in Neonatal Brains of Female Piglets Parenterally Fed with 18-Carbon n–3 Fatty Acid-Based Vegaven Compared with Fish Oil-Containing Lipid Emulsion

**DOI:** 10.1016/j.cdnut.2026.109400

**Published:** 2026-06-17

**Authors:** Eliana Lucchinetti, Phing-How Lou, Fulin Wang, Mirielle L Pauline, Mahabub Alam, Pamela R Wizzard, Zain Patel, Qiumin Tan, Patrick N Nation, Catherine J Field, Eytan Wine, Stefanie D Krämer, Paul W Wales, Justine M Turner, Michael Zaugg

**Affiliations:** 1 Department of Anesthesiology and Pain Medicine, University of Alberta, Edmonton, AB, Canada; 2 Department of Pharmacology, University of Alberta, Edmonton, AB, Canada; 3 Department of Pediatrics, University of Alberta, Edmonton, AB, Canada; 4 Department of Cell Biology, University of Alberta, Edmonton, AB, Canada; 5 Animal Pathology Services APS Ltd., Canmore, AB, Canada; 6 Faculty of Agriculture, Life and Environmental Sciences, University of Alberta, Edmonton, AB, Canada; 7 Division of Gastroenterology, Hepatology and Nutrition, The Hospital for Sick Children and University of Toronto, Toronto, ON, Canada; 8 Institute of Pharmaceutical Sciences, D-CHAB, ETH Zurich, Zurich, Switzerland; 9 Department of Surgery, Cincinnati Children’s Hospital Medical Center and University of Cincinnati, Cincinnati, OH, United States

**Keywords:** parenteral nutrition, lipopolysaccharide, insulin, energy metabolism, cAMP-response-element-binding-protein-1, c-Jun, female sex, lipid emulsions, α-linolenic acid, stearidonic acid

## Abstract

**Background:**

Vegaven, an intravenous lipid emulsion based on 18-carbon n–3 (ω–3) fatty acids, reduces the accumulation of lipopolysaccharide (LPS) in peripheral organs including the brain of parenterally fed piglets compared with fish oil-containing SMOFlipid. Inflammation may impair brain energy metabolism and growth signaling.

**Objectives:**

This study aimed to compare key brain metabolic and developmental signaling pathways with the use of different lipid emulsions for parenteral nutrition (PN).

**Methods:**

In this study, 3–4-d-old female piglets were randomly assigned to isocaloric isonitrogenous PN with Vegaven (VEGA, *N* = 10) or SMOFlipid (SMOF, *N* = 9). After 14 d of PN, plasma and tissue samples were collected.

**Results:**

LPS and tumor necrosis factor-α were lower in brain tissue samples (prefrontal cortex) of VEGA compared with SMOF. Brain pyruvate dehydrogenase activity and the ketone body β-hydroxybutyrate in both liver and brain were higher in VEGA. In contrast, there was higher phosphorylation and activation of adenosine 5′-monophosphate-activated protein kinase (p^Thr172^AMPK), a sensor of energy stress and master regulator of catabolic metabolic pathways, in brain samples of SMOF without compensatory increase in fatty acid oxidation. Anabolic signaling in the brain was higher in VEGA compared with SMOF, as indicated by higher insulin receptor substrate-1 and Akt-substrate of 160 kDa abundance, and higher phosphorylation of mammalian target of rapamycin (p^Ser2448^mTOR), essential for axon growth, dendrite arborization, and memory function. The transcription factors cAMP-response-element-binding-protein-1 (p^Ser133^CREB1) and c-Jun, critical for brain development, showed higher nuclear abundance in brain samples of VEGA compared with SMOF.

**Conclusions:**

In neonatal brains of parenterally fed female piglets, Vegaven reduced inflammation and enhanced energy metabolism and developmental signaling compared with SMOFlipid.

## Introduction

Malnutrition with extrauterine growth retardation remains a significant risk for neonates requiring parenteral nutrition (PN), specifically for preterm infants, with an estimated incidence of up to 70% [1]. Because of the rapid growth and maturation of the nervous system in the late intrauterine and early postnatal period, the brains of parenterally fed neonates are at high risk of short- and long-term neurodevelopmental impairment including neuromotor disabilities, learning and memory difficulties, and behavioral abnormalities [2]. Although many macronutrients and micronutrients are important contributors to a balanced PN regimen, intravenous lipid emulsions provide essential building blocks to form phospholipid bilayers for healthy brain growth [3]. However, when used in PN, the impact of different types of lipid emulsions on neurodevelopmental outcomes remains controversial [4,5]. Thus, not surprisingly, multidisciplinary professional groups identified the ideal fatty acid composition to achieve the best long-term effects on neurodevelopment as a high research priority in PN [6,7].

We have developed Vegaven, a novel lipid emulsion for PN, which is exclusively based on plant oils rich in 18-carbon n–3 (ω-3) fatty acids such as α-linolenic acid (ALA) and stearidonic acid (SDA), resulting in a n–6/ n–3 ratio of 1:2.5 [8]. Using a murine model of PN, Vegaven displayed anti-inflammatory, liver-protective, insulin-sensitizing, and immunity-enhancing properties with reduced gut permeability to LPS compared with soybean oil-based Intralipid [8], fish oil-based Omegaven [8], and more recently with SMOFlipid [9], a commonly used mixed-oil lipid emulsion containing fish oil [10]. Mechanistic studies suggested a critical role for lipid mediators derived from 18-carbon n–3 fatty acids in the activation of immune cells, leading to a unique immunometabolic phenotype characterized by elevated interleukin-10 (IL10) [8]. In neonatal male piglets, Vegaven has been demonstrated to be safe and equivalent to SMOFlipid with regard to weight gain, organ growth, and accretion of the 2 important fatty acids arachidonic acid (ARA) and docosahexaenoic acid (DHA) in the brain [11]. In addition, piglets that received Vegaven-based PN showed lower LPS accumulation in the brain that was accompanied by higher central insulin signaling [11], essential for proper neurodevelopment.

Building on our previous findings in male piglets, the primary aim of this study was to compare energy metabolism and neurodevelopmental signaling pathways via the insulin-like growth factor 1 receptor (IGF1R) and the insulin receptor (IR) in the brains of neonatal piglets parenterally fed with Vegaven compared with SMOFlipid. Both growth receptors signal via the same downstream adaptor protein insulin receptor substrate-1 (IRS1), and phosphorylate cAMP-response-element-binding-protein-1 (CREB1) at its serine 133 site, activating one of the critical transcription factors in brain development [12]. On the basis of our previous results [11], we hypothesized that Vegaven-fed compared with SMOFlipid-fed piglets would show higher energy metabolism and higher p^Ser133^CREB1 levels in the cortex. The second aim of this study was to corroborate the robustness of Vegaven’s immunometabolic phenotype and safety by selecting female piglets for the current investigation.

## Methods

### Study design and PN

All procedures in this study (AUP00003707) were conducted in accordance with the Canadian Council on Animal Care Guidelines and approved by the University of Alberta Animal Care and Use Committee. Female Duroc Landrace White cross-bred sow-fed piglets, 3–4-d-old, were used for total PN experiments (no enteral nutrition) according to a standardized protocol, as previously reported [11]. Female piglets were selected from each available litter and block-randomized to the study groups: SMOF (PN with SMOFlipid; *N* = 9) or VEGA (PN with Vegaven; *N* = 10). SMOFlipid was purchased from Fresenius Kabi Canada. Vegaven was manufactured in-house and subjected to rigorous quality controls, as previously described (for detailed composition see Supplemental Table 1) [8]. Isocaloric and isonitrogenous PN was initiated immediately after placing the jugular vein catheter and was delivered continuously by infusion pump for 14 d. The target caloric and nutrient intake was adjusted to daily growth with 1.1 MJ/kg/d, 16 g/kg/d amino acids, and 10 g/kg/d lipids, equivalent to a standard human infant dose of 2 g/kg/d [13]. On day 14, blood samples were collected after PN was discontinued. Bile flow was measured during terminal laparotomy, as previously described [14]. After humane euthanasia, organs including liver, spleen, bowel, and brain (prefrontal cortex) were excised, weighed, and snap frozen in liquid nitrogen for further analysis. Female sow-fed littermates of equivalent age served as reference piglets for body weight, organ growth, blood cell counts and chemistry, fatty acid and acylcarnitine profiles, liver and brain histology, and some brain metabolic measurements (*N* = 7).

### Blood cell counts and chemistry

Blood samples were sent to a veterinary laboratory for automated blood cell counts and biochemistry tests (IDEXX).

### Fatty acid profiling in phospholipid and triglyceride fractions of plasma, liver, and brain

Lipids were extracted and analyzed, as previously described [13]. For details, see Supplemental Information.

### Acylcarnitine profiling in brain tissue

Liquid Chromatography-Tandem Mass Spectrometry (LC-MS/MS) data were collected using the ionic metabolomic profiling approach (Nova Medical Testing Inc.). Data processing and statistical analyses were performed using MS-DIAL, IsoMS Pro v1.4.0 (NovaMT Inc.). Identification of 34 acylcarnitines was supported by Electrospray Ionization in positive ion mode (ESI(+)-MS/MS) spectra from authentic standards, biological samples, and in silico predictions, using the NovaMT Acylcarnitine Database v1.0 and a public lipid library. The analysis was conducted at the Metabolomics Innovative Centre (www.metabolomicscentre.ca). For further details, see Supplemental Information.

### LPS measurements in brain

LPS levels in tissue (homogenized in Phosphate-Buffered Saline (PBS)) were measured using PyroGene Recombinant Factor C endotoxin detection kit (Lonza Bioscience 50-658U; minimum detection limit of 0.005 endotoxin units/mL) [8,11].

### Cytokine measurements in tissues

Tissue IL10 was measured using R&D Systems Mouse DuoSet ELISA kit (R&D Systems DuoSet DY417-05) tested for compatibility with porcine samples. Tissue IL6 (Thermo Scientific ESIL6) and TNFα (Thermo Scientific KSC3011) were measured using porcine-specific ELISA kits. All cytokine measurements were normalized to protein concentration.

### IR β-subunit, and IRS1/2 assays

Total tissue IR β-subunit and IRS1 as well as their corresponding tyrosine phosphorylation were measured using commercial ELISA kits (Thermo Fisher Scientific #KHR9111 & KHR9121; Cell Signaling #7328 & #7133), whereas IRS2 and tyrosine phosphorylated IRS2 were measured using in-house ELISAs, as previously described [8,11].

### Quantification of glycogen content and β-hydroxybutyrate levels

Glycogen content in liver samples was measured using a commercially available kit (Abcam ab65620). β-Hydroxybutyrate levels were determined in liver and brain using BHB-Glo Ketone Body Assay (JE9500) from Promega.

### Pyruvate dehydrogenase activity

Brain mitochondrial pyruvate dehydrogenase (PDH) activity was assayed using a commercial colorimetric assay (Abcam ab109902).

### qRT-PCR

For details and primer list, see Supplemental Information.

### Immunoblotting

Total tissue homogenate was prepared using Qiagen Tissuelyser II in ice-cold (4C) Radioimmunoprecipitation Assay (RIPA) buffer, and the supernatant clarified by centrifugation at 10,000 × *g* for 5 min at 4C. Subcellular fractions (mitochondrial, nuclear, sarcolemmal) were prepared as described previously [[15], [16], [17]]. All buffers used in sample preparation were supplemented with a mixture of protease and phosphatase inhibitors. Protein concentrations were determined by DC Protein Assay (Bio-Rad Laboratories). Equal protein load was separated by SDS-PAGE and transferred to nitrocellulose membrane for probing with antibodies of interest. Immunoreactivity was visualized using Enhanced Chemiluminescence (ECL) reagent of suitable sensitivity (Western Lightning Plus Chemiluminescence Reagent by Revvity, Clarity Max Western ECL Substrate by Bio-Rad, or SuperSignal West Atto Ultimate Sensitivity Substrate by ThermoFisher Scientific), and quantified by ImageJ software (https://imagej.nih.gov/ij/). All immunoreactivity was normalized to vinculin (total tissue homogenate), Na,K-ATPase (sarcolemmal fraction) or MemCode Reversible Protein Stain (mitochondrial and nuclear fractions; Thermo Fisher Scientific 24580). For the list of antibodies, see Supplemental Information.

### Plasma insulin, glucagon, glucagon-like peptide-1, and fructosamine

Commercially available kits were used to measure plasma insulin (Mercordia 10-1200-01), glucagon (R&D systems DGCG0), glucagon-like peptide-1 (GLP-1; Thermo Scientific BMS2194), and fructosamine (Aviva Systems OKEH02616).

### Liver histology

Histology slides (hematoxylin and eosin) were obtained from 4 randomly chosen female piglets of each group and reviewed by a blinded veterinary pathologist (PNN). For further details, see Supplemental Information.

### Brain cell density studies in prefrontal cortex using immunofluorescence and confocal microscopy

For details, see Supplemental Information.

### Statistical analysis

This study was designed as direct comparison between the novel lipid emulsion Vegaven and the current standard mixed-oil fish oil-containing lipid emulsion SMOFlipid. All results were tested for normality (Shapiro–Wilk test) and summarized as mean (SD) or median (25% percentile, 75th percentile) for the indicated number (*N*) of independent experiments depending on the underlying data distribution. Differences between SMOF and VEGA were determined using a 2-tailed *t*-test or Mann–Whitney Rank Sum Test, according to the data distribution. Differences were considered significant if *P* < 0.05. SigmaPlot (version 15.1.1.26; Grafiti LLC) was used for all statistical analyses. Reference ranges from sow-fed littermates were added to some presented results (safety data including body weight, organ growth, blood cell counts and chemistry, fatty acid and acylcarnitine profiles, and some metabolic data).

## Results

### Vegaven is well tolerated and safe in parenterally fed female piglets

All piglets reached the endpoint of the study (Table 1). Sepsis was confirmed in 1 Vegaven- and 1 SMOFlipid-treated female piglet (positive blood cultures) and successfully treated. After 14 d of total PN, the change in body weight was marginally higher in VEGA compared with SMOF (Table 1). PN delivery was 7.3% higher (*P* = 0.034) in VEGA compared with SMOF (Table 1). At trial completion, liver, spleen, and brain weights were similar in both PN groups (Table 1). As expected, small bowel weight was reduced in both PN groups compared with reference female piglets indicating atrophy. Bile flows were not different between the PN groups and comparable with our historical data (Table 1) [18]. Cholestasis markers including fasting bile acid plasma concentrations, γ-glutamyl transpeptidase, and alkaline phosphatase were similar between PN groups (Supplemental Table 2). Total bilirubin plasma concentrations for both PN groups were below the upper limit of the normal range (<18 μmol/L) [19], but lower in SMOF compared with VEGA (*P* = 0.008). Blood cell counts were similar in both groups (Supplemental Table 3).

**TABLE 1:** Body weight, organ weights, and bile flow

	SMOF (*N* = 9)	VEGA (*N* = 10)	*P* value	Reference range^1^
BW day 0 (kg)	2.25 (0.12)	2.23 (0.15)	0.73	—
BW day 14 (kg)	4.71 (0.48)	4.98 (0.28)	0.15	6.0–8.5
BW change (kg)	2.46 (0.40)	2.75 (0.19)	0.06	—
Average PN delivery (% of intended dose)	84.9 (8.4)	92.2 (3.4)	0.034	—
Liver weight (g)	219.3 (35.0)	231.5 (16.4)	0.36	144–200
Spleen weight (g)	21.4 (5.2)	22.3 (5.3)	0.73	19.6–25.8
Small bowel weight (g)	97.2 (11.1)	86.7 (5.4)	0.025	190–287
Small bowel length (cm)	689 (71)	637 (49)	0.08	895–1127
Small bowel weight/length (g/cm)	0.142 (0.016)	0.137 (0.008)	0.38	0.21–0.26
Brain weight without cerebellum (g)	37.2 (1.4)	36.4 (2.0)	0.33	37.1–41.1
Brain weight with cerebellum (g)	42.4 (1.6)	41.2 (2.0)	0.20	41.8–46.3
Bile flow (μg/g liver)	9.59 (3.62)	7.78 (3.05)	0.26	6.0–13.8#

Data are expressed as mean (SD) for the SMOF and VEGA groups. *P* values refer to the comparison SMOF vs. VEGA.

Abbreviations: BW, body weight; PN, parenteral nutrition; SMOF, piglets treated with SMOFlipid-based PN for 14 d; VEGA, piglets treated with Vegaven-based PN for 14 d.

1 Values (range) from reference sow-fed littermates (*N* = 7).

### Vegaven supports adequate accretion of ARA and DHA in parenterally fed female piglet brains

In the phospholipid fractions of the brains, ARA and DHA were similar between the PN groups (Table 2). Triene-to-tetraene ratio (mead acid (MA)/ARA ratio), an index of essential fatty acid deficiency if increased, was also similar in the brain phospholipid fractions of VEGA compared with SMOF (Table 2). In the triglyceride fraction of the brain, representing backup and overflow pools of fatty acids from where the phospholipids are continuously remodeled [20], ARA was similar in VEGA compared with SMOF (Supplemental Table 4), but SDA (*P* = 0.001) and eicosatetraenoic acid (*P* = 0.003) were significantly higher in VEGA. In liver phospholipid fractions, stearic (23%), palmitic (17%), and linoleic acid (10%–13%) were dominant in both groups, but ALA, SDA, γ-linolenic acid (GLA), dihomo-GLA, EPA, and DPA were more abundant in VEGA compared with SMOF (Supplemental Table 5). As expected from the fatty acid profile of Vegaven, ARA and DHA were lower in the phospholipid fractions of liver and plasma of VEGA compared with SMOF (Supplemental Tables 5 and 6). MA/ARA ratio was lower in liver phospholipid fractions of VEGA. For additional details of fatty acid composition in liver and plasma triglyceride fractions, see Supplemental Tables 7 and 8.

**TABLE 2:** Fatty acid composition (% total fatty acids) of brain phospholipids on day 14

	SMOF (*N* = 9)	VEGA (*N* = 10)	*P* value	Reference range^1^
Myristic acid (C14:0)	0.53 (0.11)	0.59 (0.10)	0.26	0.31–0.62
Pentadecanoic acid (C15:0)	0.07 (0.06, 0.08)	0.06 (0.05, 0.07)	0.22	0.05–0.16
Palmitic acid (C16:0)	22.4 (1.76)	21.1 (1.88)	0.17	18.3–23.3
Palmitoleic acid (C16:1 n7)	0.87 (0.10)	0.92 (0.08)	0.19	0.69–1.04
Hypogeic acid (C16:1 n9)	0.96 (0.17)	0.94 (0.13)	0.77	0.54–1.12
Margaric acid (C17:0)	0.19 (0.03)	0.17 (0.01)	0.14	0.13–0.20
Stearic acid (C18:0)	23.7 (0.90)	23.1 (1.43)	0.30	20.2–24.2
Oleic acid (C18:1 n9)	16.9 (3.37)	19.2 (3.89)	0.20	13.0–22.8
Vaccenic acid (C18:1 n7)	3.63 (3.49, 4.39)	3.52 (3.32, 4.06)	0.46	3.69–4.83
Linoleic acid (C18:2 n6)	0.96 (0.10)	0.79 (0.06)	<0.001	1.00–1.61
ALA (C18:3 n3)	0.72 (0.57, 0.86)	1.01 (0.77, 1.69)	0.08	0.64–2.79
GLA (C18:3 n6)	0.065 (0.055, 0.068)	0.077 (0.066, 0.12)	0.10	0.03–0.08
SDA (C18:4 n3)	0.16 (0.15, 0.19)	0.16 (0.12, 0.19)	0.62	0.14–0.28
Arachidic acid (C20:0)	0.27 (0.21, 0.40)	0.40 (0.23, 0.48)	0.41	0.20–0.96
Eicosadienoic acid (C20:2 n6)	0.14 (0.13, 0.15)	0.11 (0.10, 0.14)	0.05	0.13–0.25
DGLA (C20:3 n6)	0.90 (0.15)	1.14 (0.21)	0.010	0.72–1.82
MA (C20:3 n9)	0.29 (0.04)	0.27 (0.06)	0.45	0.26–0.39
ARA (C20:4 n6)	9.26 (1.22)	8.82 (0.89)	0.39	8.53–11.4
ETA (C20:4 n3)	0.08 (0.06, 0.15)	0.20 (0.14, 0.32)	0.007	0.07–0.16
EPA (C20:5 n3)	0.39 (0.12)	0.57 (0.16)	0.013	0.25–0.58
Adrenic acid (C22:4 n6)	0.027 (0.02)	0.016 (0.01)	0.14	0.011–0.023
Osbond acid (C22:5 n6)	1.47 (0.45)	1.33 (0.40)	0.49	0.54–2.62
DPA (C22:5 n3)	0.58 (0.07)	1.21 (0.14)	<0.001	0.35–0.48
DHA (C22:6 n3)	9.99 (2.68)	8.58 (2.37)	0.24	5.50–10.0
Lignoceric acid (C24:0)	0.36 (0.22)	0.54 (0.31)	0.18	0.18–0.91
Nervonic acid (C24:1 n9)	3.68 (3.23, 4.32)	3.75 (3.58, 4.05)	0.68	3.75–5.77
Total SFA	47.5 (2.09)	46.0 (2.78)	0.20	42.0–48.8
Total MUFA	26.5 (4.07)	28.5 (3.99)	0.29	23.9–32.6
Total PUFA	25.4 (2.66)	24.6 (2.08)	0.45	22.6–28.0
Ratio ARA/DHA	0.86 (0.78, 1.21)	1.02 (0.84, 1.30)	0.33	1.00–1.68
Ratio MA/ARA	0.032 (0.006)	0.031 (0.009)	0.89	0.026–0.042
Ratio n6/n3	1.06 (0.18)	1.04 (0.13)	0.72	1.23–1.50

Fatty acid composition (expressed as % total fatty acids) of phospholipids isolated from brain samples. Data are expressed as mean (SD) or median (25th, 75th percentile), depending on the underlying data distribution. *P* values refer to the comparison SMOF vs. VEGA.

Abbreviations: ALA, α-linolenic acid; ARA, arachidonic acid; DGLA, dihomo-GLA; DPA, docosapentaenoic acid; ETA, eicosatetraenoic acid; GLA, γ-linolenic acid; MA, mead acid; SDA, stearidonic acid; SMOF, piglets treated with SMOFlipid-based PN for 14 d; VEGA, piglets treated with Vegaven-based PN for 14 d.

1 Values (range) from the reference sow-fed littermates (*N* = 7).

### Vegaven fosters energy metabolism and developmental signaling in neonatal brains of parenterally fed female piglets compared with SMOFlipid

**FIGURE 1:**
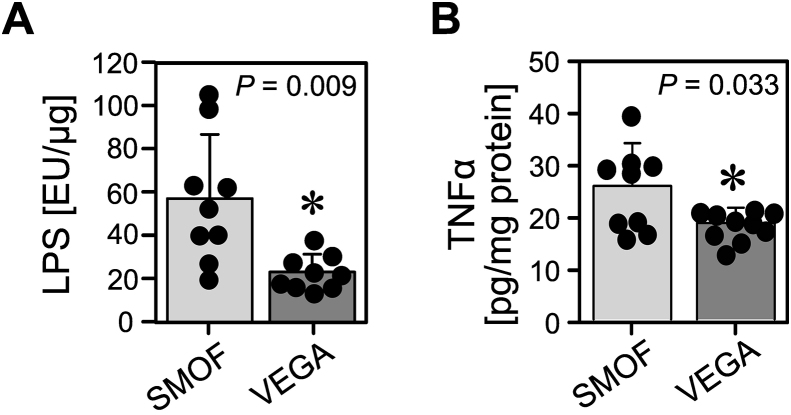
LPS and inflammation in neonatal brains of parenterally fed piglets. (A) LPS tissue concentrations as measured by an endotoxin detection testing kit. (B) TNFα levels as measured by ELISA. ∗Significantly different. Bars represent means ± SD. Dots indicate individual piglets. *N* = 9 for SMOF, *N* = 10 for VEGA. One VEGA sample was excluded from LPS analysis because it was below detection limit. PN, parenteral nutrition; SMOF, piglets treated with SMOFlipid-based PN for 14 d; VEGA, piglets treated with Vegaven-based PN for 14 d.

**FIGURE 2:**
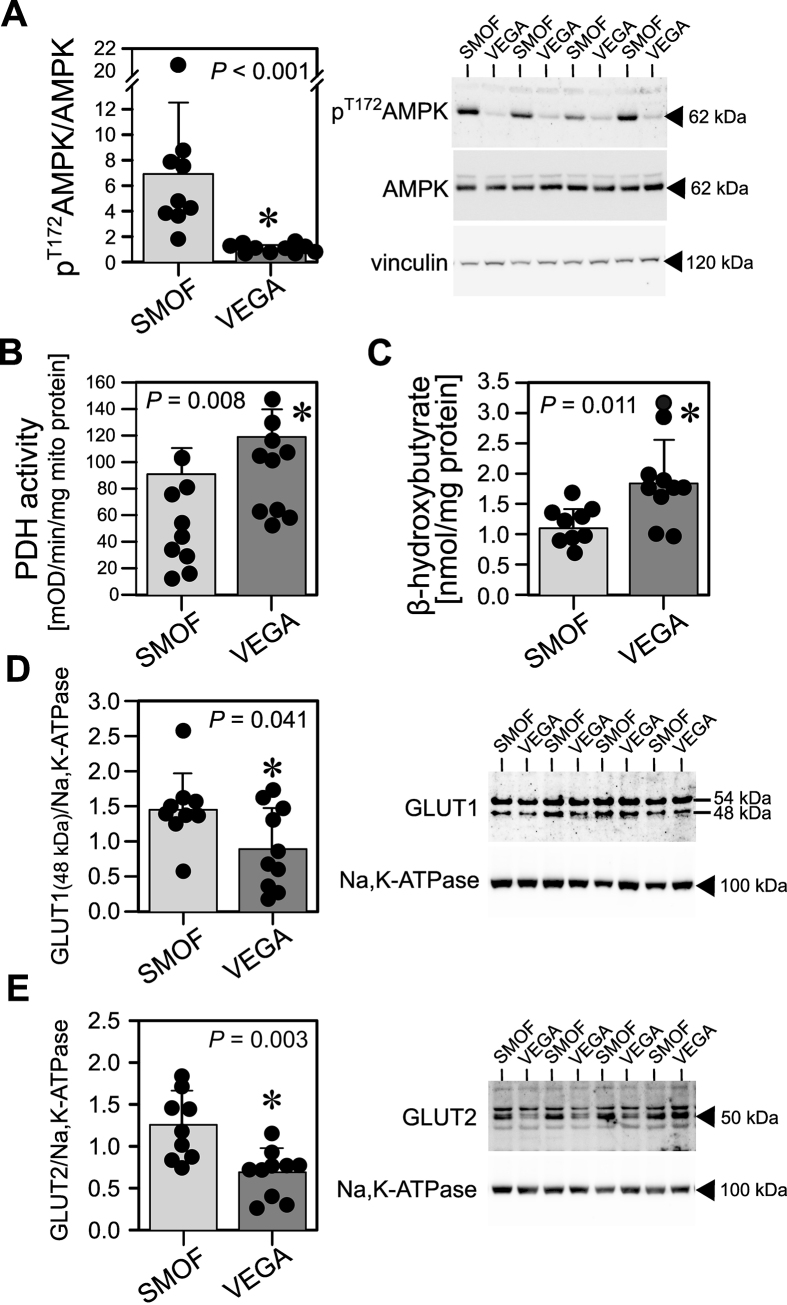
Energy metabolism in neonatal brains of parenterally fed piglets. (A) Phosphorylation (p^T172^) and activation of AMPK. There was striking AMPK activation in SMOF but not in VEGA. (B) PDH activity as measured in mitochondrial fractions. (C) Ketone body levels in brains as measured by β-hydroxybutyrate. (D) GLUT1 abundance in the plasma membrane (Na,K-ATPase was used for normalization). (E) GLUT2 abundance in plasma membrane (normalized to Na,K-ATPase). ∗Significantly different. Bars represent means ± SD. Dots indicate individual piglets. *N* = 9 for SMOF, *N* = 10 for VEGA. AMPK, AMP-activated protein kinase; GLUT, glucose transporter; PDH, pyruvate dehydrogenase; PN, parenteral nutrition; SMOF, piglets treated with SMOFlipid-based PN for 14 d; VEGA, piglets treated with Vegaven-based PN for 14 d.

LPS was significantly lower in brains of VEGA compared with SMOF (Figure 1A; *P* = 0.009), which was accompanied by lower tissue concentrations of TNFα (Figure 1B; *P* = 0.033). To explore the impact of the 2 different lipid emulsions on energy metabolism in neonatal brains, we determined AMP-activated protein kinase (AMPK) activity, a master regulator of metabolism. There was striking activation of AMPK measured by the phosphorylation of the threonine 172 residue on its catalytic α-subunit, in SMOF compared with VEGA (Figure 2A; *P* < 0.001), indicating energy stress, which we could also confirm in the male brain samples of our previous study (Supplemental Figure 1, panel A, *P* = 0.002) [11]. We also measured AMPK phosphorylation in brains of reference healthy sow-fed male and female piglets (Supplemental Figure 1, panel B). These additional measurements clearly show activation of AMPK and energy stress exclusively occurring in SMOF.

Because neurons predominantly use lactate/pyruvate from glycolysis of glucose or from the “lactate shuttle” of astrocytes to generate NADH/flavin adenine dinucleotide (FADH_2_) in the tricarboxylic acid cycle for ATP production [21], we measured mitochondrial PDH activity, which was significantly lower in SMOF compared with VEGA (Figure 2B; *P* = 0.008). In addition, the concentrations of the ketone body β-hydroxybutyrate in the brains of SMOF piglets were lower (Figure 2C; *P* = 0.011). VEGA showed similar brain PDH activity and β-hydroxybutyrate levels as in reference healthy sow-fed piglets (PDH activity: VEGA 119.0 ± 20.8 compared with reference 119.8 ± 22.1 mOD/min/mg mitochondrial protein, not significant; β-hydroxybutyrate VEGA 1.8 ± 0.7 compared with reference 1.9 ± 0.4 nmol/mg protein, not significant). Despite the reduced availability of energy substrates from glucose and ketone bodies, there was no significant compensatory increase in fatty acid oxidation (β-oxidation), as indicated by the acylcarnitine profile (Supplemental Tables 9 and 10), and no nuclear translocation of peroxisome proliferator-activated receptor gamma coactivator-1α (PGC1α) (Supplemental Figure 2). In contrast, there was increased presence of PGC1α (Supplemental Figure 2; *P* = 0.027) in mitochondrial fractions in VEGA compared with SMOF [22]. In accordance with AMPK activation in brains of SMOF piglets, there was higher glucose transporter (GLUT1; 48 kDa isoform; Figure 2D; *P* = 0.041) and GLUT2 in plasma membrane fractions (Figure 2E; *P* = 0.003). GLUT1 (endothelial 54 kDa isoform), GLUT3, and GLUT4 trafficking were similar in VEGA and SMOF (data not shown).

**FIGURE 3:**
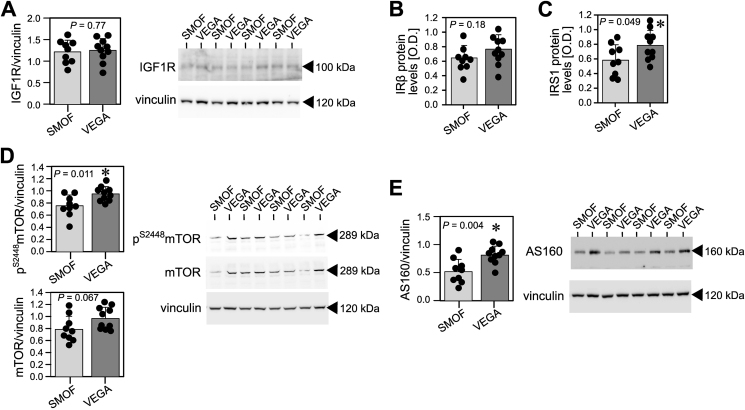
Anabolic signaling in neonatal brains of parenterally fed piglets. (A) IGF1R abundance as measured by immunoblotting. (B) IRβ abundance as measured by ELISA. (C) IRS1 abundance as measured by ELISA. Note that tyrosine phosphorylation of IRβ and IRS1 were below detection limits of the assays. (D) Phosphorylation (p^S2448^) and abundance of mTOR. (E) Akt-substrate of 160-kDa (AS160) abundance. ∗Significantly different. Bars represent means ± SD. Dots indicate individual piglets. *N* = 9 for SMOF, *N* = 10 for VEGA. IGF1R, insulin-like growth factor 1 receptor; IRβ, insulin receptor β-subunit; IRS1, insulin receptor substrate-1; mTOR, mammalian target of rapamycin; PN, parenteral nutrition; SMOF, piglets treated with SMOFlipid-based PN for 14 d; VEGA, piglets treated with Vegaven-based PN for 14 d.

**FIGURE 4:**
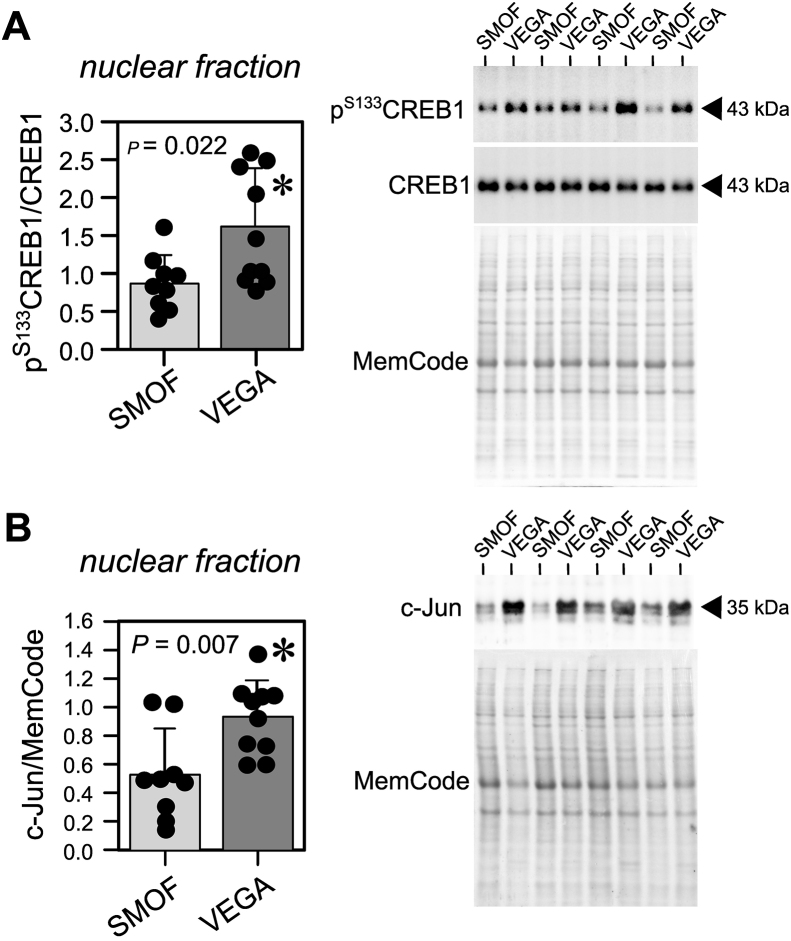
Neurodevelopmental transcription factors in neonatal brains of parenterally fed piglets. (A) Phosphorylation (p^S133^) of CREB1 in nuclear fractions. (B) Abundance of Jun proto-oncogene (c-Jun) in nuclear fractions. ∗Significantly different. Bars represent means ± SD. Dots indicate individual piglets. *N* = 9 for SMOF, *N* = 10 for VEGA. CREB1, cAMP-responsive-element-binding-protein-1; PN, parenteral nutrition; SMOF, piglets treated with SMOFlipid-based PN for 14 d; VEGA, piglets treated with Vegaven-based PN for 14 d.

IGF1R (Figure 3A) and IR (Figure 3B) were similarly expressed, but IRS1 abundance was higher in VEGA compared with SMOF (Figure 3C; *P* = 0.049). There was higher activity of the downstream serine/threonine kinase mammalian target of rapamycin (mTOR) in VEGA compared with SMOF, as indicated by higher serine 2448 phosphorylation (Figure 3D; *P =* 0.011) [23], whereas total mTOR showed a trend to higher abundance (*P =* 0.067). Moreover, the abundance of AS160, a key regulator of GLUT4 trafficking to the cell surface, was higher (Figure 3E; *P =* 0.004) in VEGA compared with SMOF [24]. CREB1, a downstream target of IGF1R/IR signaling and key transcription factor involved in neurodevelopment, synaptic plasticity, and neuroprotection [12], showed higher serine 133 phosphorylation (Figure 4A; p^Ser133^CREB1/total CREB1; *P =* 0.022) in nuclear fractions of VEGA compared with SMOF. Similarly, there was higher activation of c-Jun in VEGA compared with SMOF (Figure 4B; *P =* 0.007), another downstream target of the IGF1R/IR signaling pathways and a critical transcription factor involved in brain development [25], as measured by nuclear abundance. Immunohistochemistry of brain sections from prefrontal cortex showed no difference in the number of neurons, microglia, and astrocytes in VEGA compared with SMOF (for details, see Supplemental Figure 3).

### Vegaven’s hepatic phenotype is characterized by lower inflammation, enhanced insulin signaling, and higher ketogenesis compared with SMOFlipid

**FIGURE 5:**
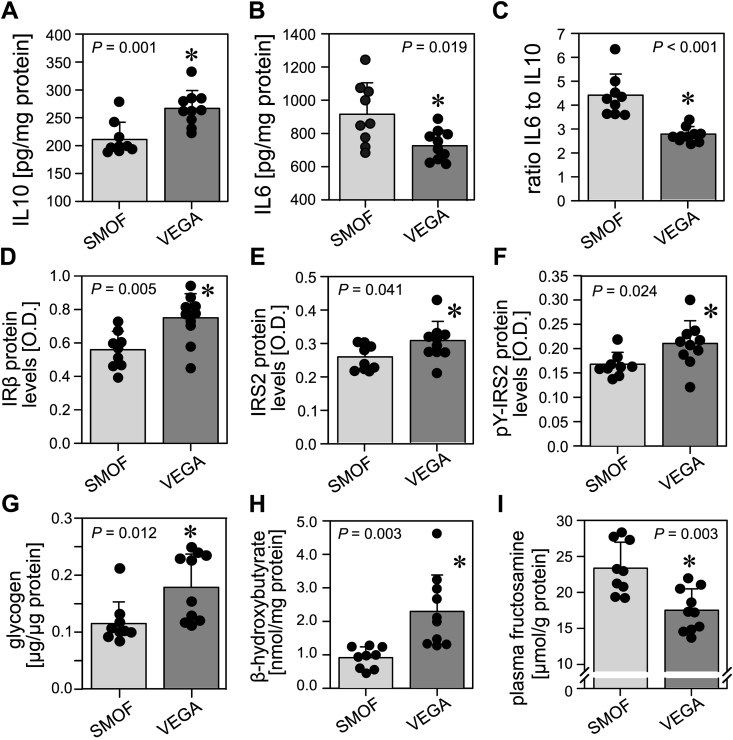
Hepatic phenotype in parenterally fed piglets: inflammation, insulin signaling, glycogen content, ketogenesis, and glycemic control. (A) IL10. (B) IL6. (C) IL-6 to IL-10 ratio. (D) IRβ abundance. (E), (F) IRS2 abundance and tyrosine phosphorylation. (G) Glycogen content in livers after 14 d of PN. (H) Ketone body levels in liver tissue as measured by β-hydroxybutyrate. (I) Fructosamine plasma levels after 14 d of parenteral PN. Values were normalized to plasma protein. Note that tyrosine phosphorylation of IRβ was below detection limit. ∗Significantly different. Bars represent mean ± SD. Dots indicate individual piglets. *N* = 9 for SMOF, *N* = 10 for VEGA. CREB1, cAMP-responsive-element-binding-protein-1; IRβ, insulin receptor β-subunit; IRS2, insulin receptor substrate-2; PN, parenteral nutrition; SMOF, piglets treated with SMOFlipid-based PN for 14 d; VEGA, piglets treated with Vegaven-based PN for 14 d.

IL10 was significantly higher (Figure 5A; *P =* 0.001) in liver tissue of VEGA compared with SMOF. In contrast, IL6 was lower (Figure 5B; *P =* 0.019) in VEGA, causing a markedly lower IL6/IL10 ratio (Figure 5C; *P* < 0.001) in VEGA compared with SMOF. IRβ (Figure 5D; *P =* 0.005), IRS2 (Figure 5E; *P =* 0.041), and pY-IRS2 (Figure 5F; *P =* 0.024) levels in liver tissue were higher in VEGA compared with SMOF. Histology sections of liver samples showed healthy liver architecture with clusters of extramedullary blood cell formation, but no infiltration of leukocytes, in both SMOF and VEGA (Supplemental Figure 4). There was also a trend toward higher glycogen vacuolation in VEGA, which was confirmed by higher glycogen measurements in VEGA compared with SMOF (Figure 5G; *P =* 0.012), indicating increased hepatic insulin sensitivity. Hepatic ketogenesis was higher in VEGA compared with SMOF (Figure 5H; *P =* 0.003). IGF1, IGF1R, and Insulin-like Growth Factor-binding Protein (IGFBP) transcripts were similarly expressed in SMOF and VEGA liver tissues (Supplemental Figure 5). Glucagon plasma concentrations were higher in SMOF (Supplemental Table 11; *P =* 0.045), which was accompanied by higher GLP-1 concentrations (Supplemental Table 11; *P =* 0.042). Insulin plasma concentrations were similar in both groups (Supplemental Table 11). Plasma fructosamine (glycated albumin) was lower in VEGA, indicating more favorable glucose control during the 14-d study period with PN (Figure 5I; *P =* 0.003).

## Discussion

Our study in parenterally-fed female piglets demonstrates that the choice of administered lipid emulsion determines the amount of LPS accumulation in neonatal brains and thus the degree of neuroinflammation, as evidenced by TNFα tissue concentrations. Neonatal LPS exposure has been demonstrated to cause dose-dependent, long-lasting adverse effects in growing brains, specifically in the prefrontal cortex and hippocampus, including demyelination, synapse loss, and cognitive decline with alterations in behavior and memory function [26,27]. We found signs of energy stress in the brain in SMOF but not VEGA, as indicated by strong AMPK activation. In the brain, neurons almost exclusively rely on glucose, lactate, pyruvate, and ketone bodies for energy production, whereas glia cells such as astrocytes and oligodendrocytes are also able to use fatty acids. In brains of SMOF piglets, PDH activity was lower, indicating impaired energy production from the end-products of glycolysis, that is, pyruvate and lactate, the latter predominantly supplied by astrocytes (“lactate shuttle”) [21]. Concomitantly, ketone bodies, another critical source of energy supplied by the liver to the neonatal brain [28], were reduced in SMOF compared with VEGA, likely caused by LPS-triggered liver inflammation and related impairment of hepatic ketogenesis [29]. Despite this reduced availability of energy substrates, there was no compensatory increase in fatty acid oxidation, as measured by acylcarnitine profiling, possibly due to the following reasons: *1*) insulin resistance in astrocytes shifting their substrate preference to glucose [30], *2*) LPS-activated microglia also switching from β-oxidation to glucose metabolism [31], and *3*) the direct inhibitory effects of LPS on β-oxidation [29] and PGC1α [32]. Although amino acid degradation and the observed increased GLUT1 and GLUT2 trafficking to the plasma membrane may have helped alleviate the energy stress [33,34], this was apparently unable to fully restore energy balance [34]. Notably, a cerebral reduction of PDH activity by 25% was reported to cause major structural defects in the developing mouse brain by affecting neuronal migration and axon growth [35]. Likewise, higher AMPK activity during brain development inhibits mTOR, the essential growth-promoting translational machinery [36] and was demonstrated to impair axon growth and arborization of dendrites [37,38]. The many catabolic effects of AMPK activation include cell cycle arrest [36] and, specifically in the context of insulin resistance, inhibition of the cholesterol synthesis and thus myelination [39], which is of particular concern in growing brains.

**FIGURE 6:**
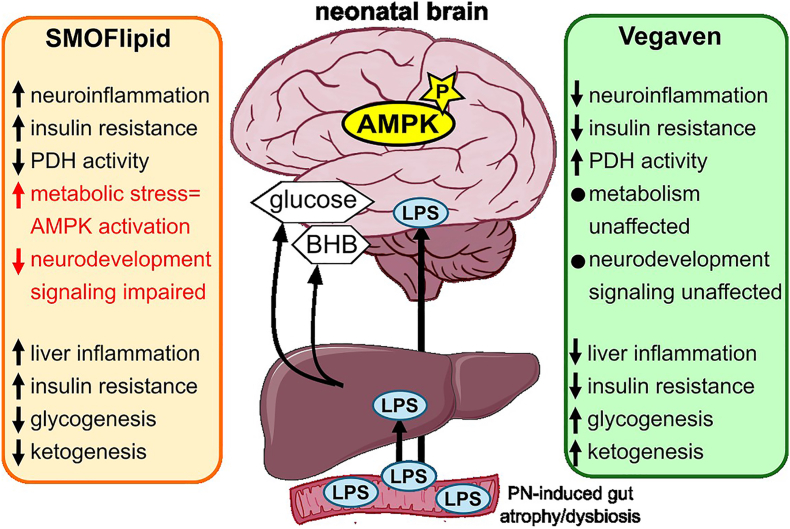
Working model. Gut atrophy and intestinal microbiome changes resulting from PN increase the release of LPS into the circulation. LPS reaches all organs including the brain and causes low-grade inflammation. This jeopardizes energy production in the neonatal brain and liver by impairing mitochondrial function, namely PDH activity, β-oxidation, and ketogenesis. Ketone bodies, namely BHB, are predominantly produced in the liver, but some ketogenesis also occurs in astrocytes. The energy stress activates the catabolic master regulator AMPK, which promotes energy production at the expense of anabolic actions such as synaptogenesis and arborization of dendrites and axons. LPS and inflammation also impair growth signaling via the IGF1R and IR by reducing the abundance of the IRS1, and key transcription factors in neurodevelopmental signaling (CREB1 and c-Jun). The use of Vegaven based on 18-carbon n–3 fatty acids but not fish oil-containing SMOFlipid (with 20/22-carbon n–3 fatty acids) in PN prevents these negative catabolic actions in growing neonatal brains. AMPK, AMP-activated protein kinase; BHB, β-hydroxybutyrate; CREB1, cAMP-responsive-element-binding-protein-1; IGF1R, insulin-like growth factor 1 receptor; IR, insulin receptor; IRS1, insulin receptor substrate-1; PDH, pyruvate dehydrogenase; PN, parenteral nutrition; SMOF, piglets treated with SMOFlipid-based PN for 14 d; VEGA, piglets treated with Vegaven-based PN for 14.

We also found higher IRS1 abundance in VEGA compared with SMOF, the joint downstream adaptor protein of 2 pivotal neurodevelopmental signaling pathways via the IR and the IGF1R [40,41]. In the presence of lower TNFα tissue concentrations, IRS1 has been shown to be less serine phosphorylated and thus functionally more active [42]. The important role of IRS1 expression in neonatal brain development has been documented in a rat study, where it promotes spine maturation, neurite branching, and dendrite arborization in hippocampal neurons [43]. IRS1 deficiency also reduces synaptic plasticity and impairs learning [44]. In our study, the positive impact of higher IRS1 abundance on downstream CREB1 activation was evident by higher CREB1 serine 133 phosphorylation, consistent with previous literature [45]. CREB1 is a key transcription factor for proper brain growth, binding to thousands of genes and modifying their expression during critical windows of brain development [12]. In line with this, n–3 PUFAs, and specifically so ALA, the dominant (>22%) fatty acid in Vegaven, were shown to upregulate CREB1 phosphorylation [46]. We also measured higher AS160 abundance and higher activity of mTOR in the brains of VEGA compared with SMOF piglets, indicators of enhanced anabolic signaling. AS160 is essential in hippocampal and some cortical neurons that are dependent on GLUT4-mediated insulin-dependent glucose uptake [24]. In our study, we observed similar GLUT4 content in membrane fractionations of VEGA and SMOF, possibly because of the strong AMPK activation in SMOF, which also increased GLUT4 trafficking. The serine/threonine kinase mTOR activates the translational machinery and is instrumental in synaptic plasticity, neurite growth, and memory function [23]. Finally, the higher nuclear abundance of c-Jun in VEGA compared with SMOF, another downstream target of the IGF1R/IR signaling pathways and a critical transcription factor involved in brain development, specifically so in the thalamus [25], corroborated these findings. Collectively, Vegaven compared with SMOFlipid supports enhanced energy production and anabolic metabolic pathways in growing brains of parenterally fed piglets (Figure 6).

Brain sizes and the number of neurons, astrocytes, and microglia were similar in VEGA and SMOF, but it is possible that subtle alterations in synapse formation and maturation, resulting from the differences in brain tissue levels of LPS and ensuing changes in energy metabolism and developmental signaling, may have occurred. Although we did not measure energy metabolism and neurodevelopmental signaling in our previous study with parenterally fed male piglets [11], that study showed even more pronounced reductions in LPS and TNFα in brain tissue of Vegaven- compared with SMOFlipid-treated piglets. We have now also confirmed exclusive and strong AMPK activation in brains of SMOFlipid-treated male piglets (Supplemental Figure 1), and thus postulate that our findings related to brain energy metabolism and brain developmental signaling are sex independent. A recent study comparing brains of SMOFlipid- compared with Intralipid-fed preterm piglets showed higher gene expression of proinflammatory cytokines in the Intralipid-treated group [47]. Similar to our study, there was no change in brain weight or the number of neurons in the prefrontal cortex [47]. Clinical studies of neurodevelopmental outcomes in PN-dependent preterm infants treated with SMOFlipid compared with Intralipid are controversial. Some studies show potential benefits with SMOFlipid (increased head circumference, but no benefit in 24-mo neurodevelopment outcome) [5], whereas others show adverse effects including myelination delay and cerebellar volume reduction [4].

Vegaven specifically acts as an immunomodulator possibly by increasing synthesis of specialized lipid mediators from 18-carbon n–3 fatty acids such as hydroxyoctadecatrienoic acids, which prime immune cells creating a cytokine microenvironment with unique anti-inflammatory, but yet immunity-enhancing properties [[48], [49], [50], [51]]. Previous studies in our PN model with mice showed that Vegaven affects the gut–microbiome–host interactions by reducing pathogenic bowel-invasive bacteria and thus the release of proinflammatory LPS into the portal vein and systemic circulation [8]. Vegaven-treated mice displayed higher anti-LPS IgG plasma titer and efficient LPS-binding protein-mediated LPS clearance in the liver as compared with mice treated with commercially available lipid emulsions [8,52]. In accordance with these concepts, our current study in parenterally fed female piglets confirms a reduction in LPS accumulation in pancreatic (Supplemental Figure 6) and brain tissues, similar to what we have observed in male piglets [11]. However, additional future experiments will help fully understand the underlying mechanisms of LPS accumulation in the brain.

Given the reported sex dimorphism in IL10-mediated immune responses [53,54], it has been unclear whether Vegaven-based PN would be equally beneficial in the female sex. Although we observed some potentially sex-related differences as compared with our previously published data in male piglets [11], this study confirms Vegaven’s beneficial biological actions in female piglets. Liver tissue cytokine levels including IL10 were generally lower in female compared with male piglets [11]; however, the IL6/IL10 ratio was equally decreased in VEGA compared with SMOF, providing evidence of lower liver inflammation. Fructosamine and insulin plasma concentrations in parenterally fed female piglets were generally lower than in male piglets [11], suggesting a more tightly controlled glucose regulation and possibly greater resilience to insulin resistance in female compared with male piglets. However, whole-body glucose control, as measured by fructosamine levels, was consistently more favorable in VEGA compared with SMOF, similar to what we reported in male piglets [11]. Interestingly, whereas hyperinsulinemia was the hallmark of a less well-controlled glucose regulation in SMOFlipid-treated male piglets [11], glucagonemia, that is, loss of insulin-mediated glucagon suppression [55], was the outstanding feature in the hormone profile of SMOF compared with VEGA. In this study, total bilirubin plasma concentrations in both groups were within normal range, but they were on average lower in SMOF compared with VEGA. It is possible that the lower total bilirubin concentrations in SMOF resulted from the higher glucagon plasma concentrations. Glucagon increases hepatic glucuronyltransferase activity and the secretory efficacy for all bilirubin pigments [56]. This is indeed supported by the close inverse correlation between glucagon and total bilirubin measurements (Supplemental Figure 7) in the absence of group differences in direct measures of cholestasis. We postulate the lower total bilirubin plasma concentrations in SMOF to be another indication of hepatic insulin resistance, supported by clinical data showing strong inverse correlation between bilirubin plasma concentrations, insulin resistance, and risk of diabetes [57,58]. Higher GLP-1 secretion in response to endotoxemia, as observed in the present study, was previously reported [59] and is mechanistically involved in the development of hyperinsulinemia. Irrespectively, robust improved whole-body glucose regulation in Vegaven-treated piglets resulted from enhanced insulin signaling in both sexes. It will be now essential to show translatability of all these observed beneficial effects from the piglet model to patients.

### Study limitations

PN delivery (percentage of intended dose) was lower in SMOF than VEGA, which may have had an impact on our metabolic and signaling results, particularly anabolic processes in the brain. However, multiple regression analysis using IRS1 a key anabolic signaling protein as dependent variable and PN delivery, group assignment, and metabolic hormones as the independent variables, identified group assignment as sole predictor of anabolic processes. To rule out the possibility that higher AMPK activation in SMOF compared with VEGA might have resulted from lower PN delivery, we also measured AMPK activity in the brains of male piglets from our previous PN study with identical study design where no intergroup difference in PN delivery occurred. Importantly and similar to the current findings, AMPK was significantly activated in brains of male piglets treated with SMOFlipid- compared with Vegaven-based PN. Finally, there was no correlation between PN delivery and AMPK activity in the brains of female piglets treated with SMOFlipid-based PN. Collectively, these observations strongly suggest that the observed metabolic and signaling differences between SMOF compared with VEGA resulted from group assignment and not from small differences in PN delivery.

## Author contributions

The authors’ responsibilities were as follows – EL, P-HL, JMT, PWW, SDK, MZ: designed research; EL, P-HL, FW, MLP, MA, PRW, ZP, QT, SDK, PNN: conducted research; EL, P-HL, FW, QT, CJF, JMT, MZ: analyzed data; MZ, JMT, PWW, JT, EW: acquired funding; EL, MZ: wrote the manuscript; MZ: primary responsible for final content; and all authors: read and approved the final manuscript.

## Data availability

Data presented and summarized in the manuscript will be made available upon request pending approval.

## Declaration of generative AI and AI-assisted technologies in the writing process

The authors declare that no generative AI or AI-assisted technologies were used in the writing of this manuscript.

## Funding

This study was funded by the Canadian Institutes of Health Research (CIHR), grant PJT190041 (MZ, JMT, PWW, EW).

## Conflict of interest

MZ has patent #PCT/EP2023/073737 pending to University of Alberta, University of Zurich, ETH Zurich. MZ has patent #EPO No. 25158072.6 pending to University of Alberta, University of Zurich, ETH Zurich. If there are other authors, they declare that they have no known competing financial interests or personal relationships that could have appeared to influence the work reported in this paper.
