# Supplementary material for: Enhanced Energy Metabolism and Developmental Signaling in Neonatal Brains of Female Piglets Parenterally Fed with 18-Carbon n–3 Fatty Acid-Based Vegaven Compared with Fish Oil-Containing Lipid Emulsion
Source: Curr Dev Nutr. 2026 Jun 17;10(7):109400. doi: 10.1016/j.cdnut.2026.109400 (PMC13356740; doi:10.1016/j.cdnut.2026.109400)
Supplement: Multimedia component 1 [file mmc1.docx]

**Online Supplementary Information**

**Enhanced energy metabolism and developmental signaling in neonatal brains of female piglets parenterally-fed with 18-carbon n-3 fatty acid-based Vegaven compared with fish oil-containing lipid emulsion**

Eliana Lucchinetti, Phing-How Lou, Fulin Wang, Mirielle L. Pauline, Mahabub Alam, Pamela R. Wizzard, Zain Patel, Qiumin Tan, Patrick N. Nation, Catherine J. Field, Eytan Wine, Stefanie D. Krämer, Paul W. Wales, Justine M. Turner, Michael Zaugg

**Supplementary Materials&Methods** Pages 2-7

**Table S1.** Composition of SMOFlipid and Vegaven Page 8

**Table S2.** Blood chemistry Page 9

**Table S3.** Blood cell counts Page 10

**Table S4**. Brain triglycerides Pages 11-12

**Table S5.** Liver phospholipids Pages 13-14

**Table S6.** Plasma phospholipids Pages 15-16

**Table S7**. Liver triglycerides Pages 17-18

**Table S8**. Plasma triglycerides Pages 19-20

**Table S9.** Lipid metabolites/acylcarnitines in brain tissue samples Page 21

**Table S10.** Brain acylcarnitine summary Page 21-22

**Table S11.** Metabolic hormones (insulin, glucagon, GLP-1) Page 22

**Figure S1.** AMPK in male piglet brains Page 23

**Figure S2.** Immunoblots of PGC-1α Page 24

**Figure S3.** Brain immunohistochemistry Page 25-26

**Figure S4.** Liver histology Page 27

**Figure S5.** Liver RT-PCR of IGF1, IGF1R, and IGFBP Page 28

**Figure S6.** Pancreas endotoxin levels Page 29

**Figure S7.** Correlation total bilirubin and glucagon Page 29-30

**Supplementary Materials&Methods**

***Fatty acid profiling in phospholipid and triglyceride fractions of plasma, liver, and brain***

Following extraction using a modified Folch method, phospholipids and triglycerides were separated by thin-layer chromatography, and fatty acids were separated, extracted and methylated, and quantitated by automatic gas-liquid chromatography (Agilent GC model 7890a; Agilent Technology). Individual fatty acids were identified as an area in the chromatogram and calculated as percentage of the total amount.

***Acylcarnitine measurements in brain tissue***

Samples were randomized before any procedures to eliminate potential technical variations from sample preparation and instrument drift. To measure concentration, 100 μL H₂O and six ceramic beads were added to each tissue sample. The samples were homogenized at 5 m/s for 15 s. An aliquot of 5 μL homogenate was used for concentration measurement with the NovaMT Sample Normalization Kit. Based on the quantification results, different volumes of H2O were added to normalize all samples to 8 mM.

For metabolite extraction from tissue, 20 μL of (normalized) sample was transferred into plastic tubes. Then, 5 μL internal standards and 80 μL LC–MS grade ACN/MeOH (1:1, v/v) were added, and the mixture was vortexed for 15 s. The homogenates were incubated at −20 °C for 30 min and centrifuged at 15,000 × g for 10 min. For each sample, 80 μL of supernatant was transferred to a new vial and dried. The dried extracts were then reconstituted in LC–MS grade MeOH/water (3:1, v/v). The LC-MS analyses were performed in negative ionization (Rapid LC-MS Analysis for HPLC Acylcarnitine Analysis Platform) on a Thermo Scientific Vanquish LC linked to Thermo Orbitrap HF Mass Spectrometer equipped with a Waters ACQUITY Premier BEH C18 VanGuard FIT Cartridge, (150 x 2.1 mm, 1.7 μm particle size). Metabolites were identified using the NovaMT Acylcarnitine database v1.0 and a public LC-MS/MS database. Identified features were normalized using internal standard ratios. Data normalization of identified features was performed by using a set of 4 stable isotope labeled internal standards at different retention time ranges. The positively and putatively identified metabolites were matched to one of the four internal standards according to retention time. Intensity ratios, i.e., intensity of each metabolite divided by intensity of the matched internal standard, were calculated for internal standard normalization. The resulting normalized intensity ratios for all identified compounds are provided in Table S9.

***Liver histology***

Following formalin fixation, liver tissue samples collected at laparatomy were paraffin-embedded. Tissue blocks were cut (3 µm) and stained with hematoxylin and eosin. A blinded veterinary pathologist (P.N.N.) independently reviewed randomly chosen liver specimens using a widely applied histology grading system for chronic hepatitis [1, 2], which was modified to evaluate parenteral nutrition-associated liver disease. The scoring system included the following 9 histologic parameters: glycogen vacuolation, intrahepatic cholestasis, necrosis, apoptosis, Kupffer cell hyperplasia, sinusoidal dilatation, portal edema, extramedullary hemopoiesis, and inflammation (leukocyte infiltration). The parameters were scored according to the scheme below.

**Glycogen vacuolation (accumulation of glycogen within hepatocytes, leading to the formation of vacuoles)**

a. Distribution:

0 = normal hepatocytes, no vacuolation

1 = glycogen vacuoles in hepatocytes, centrolobular or periportal distribution, 30% or less of lobule affected or occasional vacuolated hepatocytes in liver, no particular anatomic distribution

2 = glycogen vacuoles in hepatocyte cytoplasm, 31% to 70% of most lobules or periportal areas affected

3 = glycogen vacuoles in hepatocytes, 71% to 100% of most lobules or periportal areas affected

b. Severity

0 = normal hepatocytes

1 = occasional small intracellular glycogen vacuoles

2 = large intracellular glycogen vacuoles with normal cytoplasm in between, non-vacuolated cells also present

3 = Multiple large glycogen vacuoles completely occupying the cytoplasm (i.e,. no intervening normal cytoplasm

**Intrahepatic cholestasis**

0 = no cholestasis

1 = bile pigment in some Kuppfer cells

2 = bile pigment in Kuppfer cells and cholangioles

3 = bile pigment in Kuppfer cells, cholangioles and hepatocyte cytoplasm

**Single cell necrosis**

0 = no necrotic cells

1 = average of one necrotic cell per 400X field

2 = average of two necrotic cells per 400X field

3 = average of three or more necrotic cells per 400X field

**Apoptosis**

0 = no apoptotic bodies

1 = average of one apoptotic body per 400X field

2 = average of two apoptotic bodies per 400X field

3 = average of three or more apoptotic bodies per 400X field

**Extramedullary hemopoiesis**

This is a normal physiologic occurrence in neonatal piglets. Measured as the average of five adjacent microscope fields.

0 = no extramedullary hemopoiesis

1 = average of one or two extramedullary hemopoietic foci per 100X field

2 = average of three to five extramedullary hemopoietic foci per 100X field

3 = average of more than five extramedullary hemopoietic foci per 100X field

**All other parameters (Kupffer cell hyperplasia, sinusoidal dilatation, portal edema, inflammation)**

0 = normal

1 = mild/moderate (less than half lobule affected)

2 = severe (more than half lobule affected)

**Additional references (liver histology)**

1. Scheuer PJ. Classification of chronic viral hepatitis: a need for reassessment. J Hepatol 1991; 13: 372–374.
2. Hua Z, Sergi C, Nation PN et al. Hepatic ultrastructure in a neonatal piglet model of intestinal failure-associated liver disease (IFALD). J Electron Microsc. 2012; 61(3): 179-186.

***Antibodies used for immunoblotting***

| **Primary Antibodies** | **Molecular weight** | **Source** | **Catalog #** |
| --- | --- | --- | --- |
| phospho-AMPKα (Thr172) | 62 kDa | Proteintech Group | 80209-6-RR |
| AMPKα Polyclonal Antibody | 62 kDa | Proteintech Group | 10929-2-AP |
| phospho-AS160 (Thr642) | 160 kDa | Cell Signaling | 8881 |
| AS160 Monoclonal Antibody | 160 kDa | Cell Signaling | 2670 |
| phospho-CREB1 (Ser133) | approx. 43 kDa | Proteintech Group | 28792-1-AP |
| CREB1 Monoclonal Antibody | approx. 43 kDa | Proteintech Group | 67927-1-Ig |
| GLUT1 Monoclonal Antibody | 48 kDa/54 kDa | Proteintech Group | 66290-1-Ig |
| GLUT2 | 50 kDa | Millipore | 07-1402-I |
| GLUT3 Polyclonal Antibody | 45 kDa | Proteintech Group | 20403-1-AP |
| GLUT4 Monoclonal Antibody | 55 kDa | Cell Signaling | 2213 |
| IGF1R/CD221 Polyclonal Antibody | approx. 100 kDa | Proteintech Group | 20254-1-AP |
| c-Jun Polyclonal Antibody | 39 kDa | Proteintech Group | 24909-1-AP |
| phospho-mTOR (Ser2448) | 289 kDa | Cell Signaling | 2971 |
| mTOR Antibody | 289 kDa | Cell Signaling | 2972 |
| Na,K-ATPase α1 | 100 kDa | Cell Signaling | 3010 |
| PGC1a Monoclonal Antibody | 90-100 kDa | Proteintech Group | 66369-1-Ig |
| Vinculin | 120 kDa | Abcam | ab18058 |

***Reverse transcription-quantitative real-time polymerase chain reaction (RT-qPCR)***

RNA was extracted from liver tissue samples using TRIzol^TM^ Reagent (ThermoFisher, USA) following manufacturer’s instructions. RT-qPCR reactions were set up using the Luna® Universal One-Step RT-qPCR Kit (New England Biolabs, Inc. Whitby, Canada) and performed in a LightCycler 480 (Roche, Switzerland). The threshold cycle (Ct) values were determined using the LightCycler® 480 SW 1.5.1 (Roche, Switzerland) software with the second derivative method. Relative gene expression was calculated using the ΔΔCt method, normalized to the control group and using TATA-binding protein gene (*TBP*) as the reference gene. The primer information for the genes of interest, namely insulin like growth factor 1 (*IGF1*), insulin like growth factor 1 receptor (*IGF1R*), and insulin-like growth factor-binding protein (*IGFBP3*) is listed in the following table.

| Gene | RefSeq ID | Primer sequence (5' -> 3') | | Product size |
| --- | --- | --- | --- | --- |
| *IGF1* | DQ784687 | Forward: | TTTCAACAAGCCCACAGGGT | 102 |
|  |  | Reverse: | TCCAGCCTCCTCAGATCACA |  |
| *IGF1R* | AB003362.2 | Forward: | CATTGACATCCGCAACGACTA | 81 |
|  |  | Reverse: | AGCAGGATGTGGAGGTAGCC |  |
| *IGFBP3* | NM_001005156.1 | Forward: | GACACGCTGAACCACCTCA | 151 |
|  |  | Reverse: | CGTACTTATCCACGCACCAG |  |
| *TBP* | XM_021085488.1 | Forward: | AGGAGTTCTGTAGGGTCGGG | 86 |
|  |  | Reverse: | CTACTACCGGCAGGCGAAAA |  |

***Brain cell density studies in prefrontal cortex using immunofluorescence and confocal microscopy***

Prefrontal cortical tissue was fixed with 10% formalin overnight and subsequently transferred to cryoprotective 15% and 30% sucrose, each for 24 h. The tissue was embedded in Tissue-Tek OCT compound, cryosections (40 µM) were cut using a cryostat, transferred to microscope slides, and air-dried before undergoing staining procedures. Sections were permeabilized with Triton X-100, and subjected to primary and secondary antibodies. After autofluorescence quenching, the sections were counterstained with DAPI and mounted. A laser-scanning confocal microscope (Zeiss LSM 700) was used to obtain images. Two cortical columns were imaged and tiled and z-stacked images were acquired for each sample. Cell counting and area measurements were performed using Fiji ImageJ software (https://imagej.net/software/fiji/). The following primary antibodies were used for immunofluorescence staining: goat anti-IBA1 (ionized calcium-binding adapter molecule 1, microglia marker) (1:500, NB100-1028, Bio-Techne Canada); rabbit anti-GFAP (glial fibrillary acidic protein, astrocyte marker) (1:500, Z033429-2, DAKO/Agilent Technologies Canada Inc.); mouse anti-SATB1+SATB2 (special AT-rich sequence binding protein 1 and 2, neuronal markers) (1:500, ab51502, Abcam Inc.). The secondary antibodies were: donkey anti-goat IgG (H+L) Alexa Fluor 555 (1:1000; A-21432, Thermo Fisher Scientific); donkey anti-rabbit IgG (H+L) Alexa Fluor 488 (1:1000; A-21206, Thermo Fisher Scientific); and donkey anti-mouse IgG (H+L) Alexa Fluor 647 (1:1000; A-31571, Thermo Fisher Scientific).

**Table S1.** Composition of the emulsions used

|  | **SMOFlipid 20%** | **Vegaven 20%** |
| --- | --- | --- |
| ***Oil source, g/100mL*** | | |
| Soybean oil | 6 g (30%) | 0 g |
| Olive oil | 5 g (25%) | 5 g (25%) |
| Coconut oil | 6 g (30%) | 5 g (25%) |
| Fish oil | 3 g (15%) | 0 g |
| *Buglossoides arvensis* oil | 0 g | 10 g (50%) |
| ***Nutritionally important fatty acids, %-weight of total fatty acids*** | | |
| Linoleic acid (C18:2 n6) | 18.6% | 9.1% |
| α-Linolenic acid (C18:3 n3) | 2.4% | 21.6% |
| γ-Linolenic acid (C18:3 n6) | not reported | 3.5% |
| Stearidonic acid (C18:4 n3) | 0.4% | 10% |
| Eicosapentaenoic acid (EPA; C20:5 n3) | 2.4% | -- |
| Docosahexaenoic acid (DHA; C22:6 n3) | 2.2% | -- |
| Arachidonic acid (ARA, C20:4 n6) | 0.5% | -- |
| PUFA g% | 29% | 44% |
| MUFA g% | 30% | 27% |
| SFA g% | 41% | 29% |
| n-6 fatty acids g% | 19.1% | 13% |
| n-3 fatty acids g% | 7.7% | 32% |
| n-6 to n-3 ratio | 2.5:1 | 1:2.5 |

SMOFlipid contains 1.162-0.225 mg/mL *all rac*-α-tocopherol as per drug information sheet. Vegaven contains 0.16 mg/mL D-α-tocopherol. The mean total phytosterol concentration in SMOFlipid is 179±10 µg/mL. Total phytosterol concentration in Vegaven is 373±12 µg/mL.

Abbreviations: PUFA, polyunsaturated fatty acids; MUFA, monounsaturated fatty acids; SFA, saturated fatty acids.

**Table S2.** Blood chemistry

|  | **SMOF (N=9)** | **VEGA (N=10)** | **p-value** | **reference range$** |
| --- | --- | --- | --- | --- |
| glucose, mmol/L | 3.59 [1.30] | 2.69 [1.30] | 0.15 | 5.5 – 7.9 |
| Blood Urea Nitrogen (BUN) (mmol/L) | 1.90 [1.45, 2.15] | 1.90 [1.75, 2.33] | 0.57 | 1.2 – 3.5 |
| creatinine (μmol/L) | 32.6 [8.3] | 33.8 [5.6] | 0.70 | 56 – 75 |
| sodium (mmol/L) | 139.8 [2.1] | 139.1 [3.4] | 0.61 | 139 – 143 |
| potassium (mmol/L) | 4.67 [0.38] | 4.67 [0.37] | 0.99 | 3.8 – 4.5 |
| Na/K ratio | 30.2 [2.8] | 30.0 [3.0] | 0.91 | 32 – 38 |
| chloride (mmol/L) | 106.2 [2.0] | 107.1 [2.3] | 0.39 | 102 – 107 |
| phosphorus (mmol/L) | 2.18 [0.28] | 1.97 [0.16] | 0.06 | 2.9 – 3.3 |
| calcium (mmol/L) | 2.77 [0.11] | 2.82 [0.12] | 0.34 | 2.6 – 2.8 |
| anion gap | 15.0 [11.5, 16.0] | 13.5 [10.8, 15.0] | 0.34 | 10 – 18 |
| TCO_2_ (bicarbonate) (mmol/L) | 24.4 [1.3] | 23.7 [2.5] | 0.44 | 24 – 30 |
| creatine kinase (IU/L) | 614 [493, 1315] | 441 [363, 569] | 0.07 | 187 – 554 |
| osmolality [mmol/kg] | 274.4 [3.4] | 272.3 [4.6] | 0.27 | 274 – 282 |
| protein (g/L) | 38.9 [2.9] | 41.1 [2.0] | 0.08 | 43 – 47 |
| albumin (g/L) | 17.3 [1.7] | 17.5 [1.8] | 0.84 | 24 – 33 |
| globulin (g/L) | 21.6 [2.4] | 23.6 [2.0] | 0.06 | 13 – 19 |
| albumin:globulin ratio | 0.80 [0.12] | 0.75 [0.15] | 0.44 | 1.3 – 2.5 |
| triglycerides (mmol/L) | 0.35 [0.30, 0.43] | 0.39 [0.32, 0.41] | 0.74 | 0.28 – 0.48 |
| ALP (IU/L) | 585.2 [186.0] | 693.6 [137.6] | 0.16 | 573 – 849 |
| AST (IU/L) | 34.7 [11.3] | 37.9 [10.6] | 0.53 | 22 – 28 |
| ALT (IU/L) | 12.7 [1.6] | 13.3 [2.5] | 0.52 | 22 – 29 |
| GGT (IU/L) | 58.2 [13.6] | 76.8 [32.5] | 0.13 | 21 – 40 |
| total bilirubin (μmol/L) | 6.56 [3.42] | 11.5 [3.73] | **0.008** | 2.4 – 4.7# |
| total bile acids (µmol/L) | 13.4 [6.8, 22.6] | 13.2 [7.5, 18.8] | 0.87 | 7.6 – 20.7 |

Data are expressed as mean [SD] or median [25th,75th percentile], depending on the underlying data distribution. P-values refer to the comparison SMOF vs. VEGA.

$, Values (range) from reference sow-fed littermates (N=7). #, Reference range for total bilirubin plasma concentrations in healthy piglets is 2.0 – 18.0 µmol/L.

Abbreviations: ALP, alkaline phosphatase; ALT, alanine transaminase; AST, aspartate aminotransferase; GGT, γ-glutamyl transpeptidase; PN, parenteral nutrition; SMOF, piglets treated with SMOFlipid-based PN for 14 days; VEGA, piglets treated with Vegaven-based PN for 14 days.

**Table S3.** Blood cell count

|  | **SMOF (N=9)** | **VEGA (N=10)** | **p-value** | **reference range$** |
| --- | --- | --- | --- | --- |
| WBC (10^9^/L) | 8.4 [7.4, 13.7] | 8.1 [7.1, 10.5] | 0.60 | 4.9 – 12 |
| RBC (10^12^/L) | 5.44 [0.51] | 5.08 [0.36] | 0.10 | 4.6 – 6.0 |
| hemoglobin (g/L) | 95.4 [6.6] | 89.4 [6.9] | 0.08 | 96 – 115 |
| hematocrit (L/L) | 0.34 [0.02] | 0.33 [0.02] | 0.16 | 0.33 – 0.39 |
| MCV (fL) | 63.0 [5.3] | 65.3 [3.2] | 0.26 | 63.2 – 78.7 |
| MCH (pg/cell) | 17.6 [0.9] | 17.8 [0.9] | 0.67 | 18.8 – 22.8 |
| MCHC (g/L) | 279.9 [14.0] | 271.8 [7.0] | 0.12 | 289.2 – 315.2 |
| RDW,% | 16.7 [1.3] | 17.3 [1.1] | 0.38 | 17.2 – 21.2 |
| reticulocyte count (%) | 10.7 [4.7] | 12.1 [5.7] | 0.56 | 5.8 – 14 |
| reticulocytes (10^3^/µL) | 579.3 [252.3] | 539.7 [191.6] | 0.71 | 296 – 840 |
| nucleated RBC (/100 RBC) | 11.0 [9.1] | 19.8 [14.7] | 0.13 | 2 – 9 |
| neutrophils (10^9^/L) | 4.1 [3.6, 8.3] | 4.5 [3.4, 5.6] | 0.97 | 1.6 – 8.4 |
| lymphocytes (10^9^/L) | 3.32 [1.03] | 3.00 [0.99] | 0.50 | 2.3 – 5.1 |
| monocytes (10^9^/L) | 0.83 [0.47] | 0.76 [0.34] | 0.71 | 0.3 – 0.6 |
| platelets (10^9^/L) | 449.3 [136.7] | 524.3 [79.9] | 0.18 | 352 – 482 |

Data are expressed as mean [SD] or median [25th,75th percentile], depending on the underlying data distribution. P-values refer to the comparison SMOF vs. VEGA.

$, Values (range) from the reference sow-fed littermates (N=7).

Abbreviations: WBC, white blood cells; RBC, red blood cells; MCV, mean corpuscular volume; MCH, mean corpuscular hemoglobin; MCHC, mean corpuscular hemoglobin concentration; RWD, red cell distribution width; PN, parenteral nutrition; SMOF, piglets treated with SMOFlipid-based PN for 14 days; VEGA, piglets treated with Vegaven-based PN for 14 days.

**Table S4.** Fatty acid composition of brain triglycerides (% total fatty acids) on day 14

|  | **SMOF (N=9)** | **VEGA (N=10)** | **p-value** | **reference range$** |
| --- | --- | --- | --- | --- |
| Myristic acid (C14:0) | 0.88 [0.16] | 1.12 [0.32] | 0.05 | 0.69 – 1.79 |
| Pentadecanoic acid (C15:0) | 0.20 [0.08] | 0.22 [0.10] | 0.57 | 0.15 – 0.33 |
| Palmitic acid (C16:0) | 31.1 [5.37] | 31.0 [5.86] | 0.97 | 30.8 – 37.3 |
| Palmitoleic acid (C16:1 n7) | 0.85 [0.71,0.94] | 0.96 [0.85,1.15] | 0.10 | 1.08 – 2.94 |
| Hypogeic acid (C16:1 n9) | 0.88 [0.15] | 0.91 [0.17] | 0.74 | 0.69 – 1.26 |
| Margaric acid (C17:0) | 0.37 [0.08] | 0.39 [0.11] | 0.59 | 0.32 – 0.57 |
| Stearic acid (C18:0) | 27.5 [4.74] | 26.4 [3.60] | 0.57 | 19.2 – 26.0 |
| Oleic acid (C18:1 n9) | 14.2 [4.44] | 14.8 [3.95] | 0.76 | 11.1 – 22.2 |
| Vaccenic acid (C18:1 n7) | 2.89 [0.77] | 2.70 [0.74] | 0.59 | 2.79 – 3.78 |
| Linoleic acid (C18:2 n6) | 2.95 [1.83,3.01] | 2.02 [1.68,2.87] | 0.68 | 2.35 – 5.09 |
| α-linolenic acid (ALA; C18:3 n3) | 0.72 [0.66,1.13] | 1.06 [0.79,1.96] | 0.07 | 0.89 – 1.68 |
| γ-linolenic acid (GLA; C18:3 n6) | 0.16 [0.08,0.24] | 0.22 [0.13,0.43] | 0.07 | 0.13 – 0.30 |
| Stearidonic acid (SDA; C18:4 n3) | 0.18 [0.16,0.21] | 0.32 [0.24,0.41] | **0.001** | 0.16 – 0.33 |
| Arachidic acid (C20:0) | 0.49 [0.13] | 0.53 [0.13] | 0.60 | 0.30 – 0.82 |
| Eicosadienoic acid (C20:2 n6) | 0.18 [0.05] | 0.17 [0.05] | 0.53 | 0.15 – 0.22 |
| Dihomo-γ-linolenic acid (DGLA; C20:3 n6) | 0.85 [0.26] | 1.09 [0.47] | 0.20 | 0.54 – 1.28 |
| Mead acid (MA; C20:3 n9) | 0.23 [0.04] | 0.22 [0.05] | 0.54 | 0.17 – 0.39 |
| Arachidonic acid (ARA, C20:4 n6) | 6.40 [1.83] | 5.79 [1.73] | 0.46 | 4.54 – 8.85 |
| Eicosatetraenoic acid (ETA; C20:4 n3) | 0.25 [0.13] | 0.59 [0.28] | **0.003** | 0.12 – 0.42 |
| Eicosapentaenoic acid (EPA; C20:5 n3) | 0.44 [0.40,0.54] | 0.48 [0.34,0.94] | 0.87 | 0.19 – 0.63 |
| Osbond acid (C22:5 n6) | 0.50 [0.46,0.90] | 0.44 [0.34,0.79] | 0.25 | 0.37 – 1.01 |
| Docosapentaenoic acid (DPA; C22:5 n3) | 0.59 [0.16] | 0.67 [0.31] | 0.47 | 0.21 – 0.47 |
| Docosahexaenoic acid (DHA; C22:6 n3) | 4.55 [1.99] | 3.84 [2.06] | 0.46 | 1.80 – 3.87 |
| Lignoceric acid (C24:0) | 0.28 [0.06] | 0.25 [0.06] | 0.43 | 0.16 – 0.31 |
| Nervonic acid (C24:1 n9) | 1.20 [0.90,2.40] | 0.86 [0.75,2.10] | 0.33 | 0.68 – 2.00 |
|  |  |  |  |  |
| Total SFA | 60.8 [10.0] | 59.9 [9.03] | 0.85 | 54.0 – 65.0 |
| Total MUFA | 20.5 [5.83] | 20.9 [4.88] | 0.85 | 17.3 – 30.3 |
| Total PUFA | 18.4 [4.58] | 18.7 [4.98] | 0.87 | 14.6 – 20.4 |
| Ratio ARA/DHA | 1.52 [0.32] | 1.68 [0.39] | 0.36 | 1.67 – 2.60 |
| Ratio MA/ARA | 0.03 [0.03,0.04] | 0.04 [0.03,0.05] | 0.22 | 0.038 – 0.046 |
| Ratio n6/n3 | 1.60 [0.25] | 1.41 [0.31] | 0.16 | 1.81 – 2.79 |

Fatty acid composition (expressed as % total fatty acids) of triglycerides isolated from brain samples. Data are expressed as mean [SD] or median [25th,75th percentile], depending on the underlying data distribution. P-values refer to the comparison SMOF vs. VEGA.

$, Values (range) from the reference sow-fed littermates (N=7).

Abbreviations: PN, parenteral nutrition; SMOF, piglets treated with SMOFlipid-based PN for 14 days; VEGA, piglets treated with Vegaven-based PN for 14 days.

**Table S5.** Fatty acid composition (% total fatty acids) of liver phospholipids on day 14

|  | **SMOF (N=9)** | **VEGA (N=10)** | **p-value** | **reference range$** |
| --- | --- | --- | --- | --- |
| Lauric acid (C12:0) | 0.013 [0.003] | 0.054 [0.013] | **<0.001** | 0.008 – 0.026 |
| Myristic acid (C14:0) | 0.45 [0.10] | 0.91 [0.13] | **<0.001** | 0.15 – 0.27 |
| Pentadecanoic acid (C15:0) | 0.082 [0.08,0.09] | 0.050 [0.047,0.06] | **0.001** | 0.043 – 0.052 |
| Palmitic acid (C16:0) | 16.9 [0.84] | 17.4 [0.80] | 0.18 | 17.9 – 18.5 |
| Palmitoleic acid (C16:1 n7) | 0.86 [0.11] | 0.78 [0.19] | 0.30 | 1.34 – 1.83 |
| Hypogeic acid (C16:1 n9) | 0.47 [0.07] | 0.50 [0.10] | 0.58 | 0.14 – 0.20 |
| Margaric acid (C17:0) | 0.38 [0.05] | 0.27 [0.04] | **<0.001** | 0.20 – 0.28 |
| Stearic acid (C18:0) | 23.9 [0.66] | 22.6 [0.78] | **0.001** | 25.8 – 27.2 |
| Oleic acid (C18:1 n9) | 13.0 [0.75] | 14.9 [0.74] | **<0.001** | 7.61 – 8.22 |
| Cis-vaccenic acid (C18:1 n7) | 1.81 [0.19] | 1.27 [0.23] | **<0.001** | 1.99 – 2.21 |
| Linoleic acid (C18:2 n6) | 13.5 [0.72] | 9.62 [0.47] | **<0.001** | 12.6 – 14.4 |
| α-linolenic acid (ALA; C18:3 n3) | 0.32 [0.05] | 1.46 [0.28] | **<0.001** | 0.31 – 0.45 |
| γ-linolenic acid (GLA; C18:3 n6) | 0.15 [0.12,0.17] | 0.54 [0.51,0.60] | **<0.001** | 0.13 – 0.16 |
| Stearidonic acid (SDA; C18:4 n3) | 0.098 [0.07,0.11] | 0.13 [0.10,0.16] | **0.011** | 0.027 – 0.100 |
| Arachidic acid (C20:0) | 0.28 [0.03] | 0.26 [0.04] | 0.18 | 0.18 – 0.28 |
| Eicosadienoic acid (C20:2 n6) | 0.35 [0.33,0.38] | 0.16 [0.15,0.17] | **<0.001** | 0.23 – 0.31 |
| Dihomo-γ-linolenic acid (DGLA; C20:3 n6) | 2.81 [0.41] | 5.10 [0.56] | **<0.001** | 0.91 – 1.30 |
| Mead acid (MA; C20:3 n9) | 0.23 [0.04] | 0.13 [0.02] | **<0.001** | 0.28 – 0.39 |
| Arachidonic acid (ARA, C20:4 n6) | 10.2 [0.60] | 8.68 [0.42] | **<0.001** | 15.4 – 17.1 |
| Eicosatetraenoic acid (ETA; C20:4 n3) | 0.20 [0.15,0.28] | 2.75 [2.37,3.06] | **<0.001** | 0.036 – 0.054 |
| Eicosapentaenoic acid (EPA; C20:5 n3) | 2.03 [0.30] | 3.70 [0.75] | **<0.001** | 0.27 – 0.48 |
| Osbond acid (C22:5 n6) | 0.228 [0.03] | 0.234 [0.03] | 0.73 | 0.39 – 0.58 |
| Docosapentaenoic acid (DPA; C22:5 n3) | 2.29 [0.34] | 2.70 [0.39] | **0.025** | 2.11 – 2.42 |
| Docosahexaenoic acid (DHA; C22:6 n3) | 8.18 [0.39] | 4.83 [0.40] | **<0.001** | 5.19 – 6.05 |
| Lignoceric acid (C24:0) | 0.49 [0.06] | 0.32 [0.07] | **<0.001** | 0.32 – 0.60 |
| Nervonic acid (C24:1 n9) | 0.23 [0.21,0.26] | 0.15 [0.13,0.17] | **<0.001** | 0.47 – 0.65 |
|  |  |  |  |  |
| Total SFA | 42.5 [0.77] | 41.9 [0.66] | 0.10 | 41.3 – 43.9 |
| Total MUFA | 16.5 [0.82] | 17.6 [0.93] | **0.010** | 15.3 – 17.6 |
| Total PUFA | 40.6 [1.32] | 39.9 [0.81] | 0.18 | 38.4 – 42.2 |
| Ratio ARA/DHA | 1.28 [1.19,1307] | 1.78 [1.72,1.88] | **<0.001** | 2.75 – 3.12 |
| Ratio MA/ARA | 0.022 [0.004] | 0.015 [0.002] | **<0.001** | 0.018 – 0.025 |
| Ratio n6/n3 | 2.09 [0.12] | 1.58 [0.11] | **<0.001** | 3.47 – 3.99 |

Fatty acid composition (expressed as % total fatty acids) of phospholipids isolated from liver samples. Data are expressed as mean [SD] or median [25th,75th percentile], depending on the underlying data distribution. P-values refer to the comparison SMOF vs. VEGA.

$, Values (range) from the reference sow-fed littermates (N=7).

Abbreviations: PN, parenteral nutrition; SMOF, piglets treated with SMOFlipid-based PN for 14 days; VEGA, piglets treated with Vegaven-based PN for 14 days.

**Table S6.** Fatty acid composition (% total fatty acids) of plasma phospholipids on day 14

|  | **SMOF (N=9)** | **VEGA (N=10)** | **p-value** | **reference range$** |
| --- | --- | --- | --- | --- |
| Capric acid (C10:0) | 0.036 [0.014] | 0.022 [0.004] | **0.020** | 0.005 – 0.022 |
| Lauric acid (C12:0) | 0.043 [0.036,0.061] | 0.052 [0.048,0.062] | 0.12 | 0.015 – 0.033 |
| Myristic acid (C14:0) | 0.45 [0.06] | 0.77 [0.08] | **<0.001** | 0.22 – 0.34 |
| Pentadecanoic acid (C15:0) | 0.12 [0.11,0.14] | 0.08 [0.07,0.09] | **0.003** | 0.05 – 0.09 |
| Palmitic acid (C16:0) | 28.8 [1.79] | 29.1 [1.38] | 0.68 | 26.0 – 30.5 |
| Palmitoleic acid (C16:1 n7) | 0.61 [0.07] | 0.58 [0.05] | 0.18 | 1.25 – 1.70 |
| Hypogeic acid (C16:1 n9) | 0.50 [0.04] | 0.41 [0.06] | **0.002** | 0.11 – 0.24 |
| Margaric acid (C17:0) | 0.36 [0.33,0.41] | 0.26 [0.24,0.27] | **0.003** | 0.24 – 0.35 |
| Stearic acid (C18:0) | 20.6 [0.98] | 19.2 [1.06] | **0.010** | 19.1 – 21.0 |
| Oleic acid (C18:1 n9) | 17.0 [1.02] | 21.3 [1.45] | **<0.001** | 10.8 – 11.9 |
| Vaccenic acid (C18:1 n7) | 2.10 [0.38] | 1.93 [0.10] | 0.23 | 2.20 – 2.46 |
| Linoleic acid (C18:2 n6) | 12.1 [11.6,12.4] | 10.4 [10.1,10.5] | **<0.001** | 20.9 – 23.4 |
| α-linolenic acid (ALA; C18:3 n3) | 0.31 [0.28,0.32] | 0.84 [0.81,0.88] | **<0.001** | 0.71 – 1.00 |
| γ-linolenic acid (GLA; C18:3 n6) | 0.07 [0.05,0.10] | 0.17 [0.16,0.18] | **<0.001** | 0.06 – 0.12 |
| Stearidonic acid (SDA; C18:4 n3) | 0.11 [0.014] | 0.16 [0.022] | **<0.001** | 0.05 – 0.18 |
| Arachidic acid (C20:0) | 0.73 [0.22] | 0.39 [0.06] | **0.002** | 0.18 – 0.30 |
| Eicosadienoic acid (C20:2 n6) | 0.33 [0.030] | 0.19 [0.016] | **<0.001** | 0.21 – 0.25 |
| Dihomo-γ-linolenic acid (DGLA; C20:3 n6) | 2.11 [0.37] | 2.55 [0.34] | **0.015** | 0.56 – 0.77 |
| Mead acid (MA; C20:3 n9) | 0.15 [0.017] | 0.11 [0.019] | **<0.001** | 0.15 – 0.26 |
| Arachidonic acid (ARA, C20:4 n6) | 5.62 [0.67] | 4.65 [0.46] | **0.002** | 5.90 – 8.89 |
| Eicosatetraenoic acid (ETA; C20:4 n3) | 0.11 [0.09,0.13] | 1.06 [0.98,1.33] | **<0.001** | 0.014 – 0.046 |
| Eicosapentaenoic acid (EPA; C20:5 n3) | 0.99 [0.10] | 1.26 [0.30] | **0.023** | 0.24 – 0.40 |
| Adrenic acid (C22:4 n6) | 0.058 [0.012] | 0.021 [0.004] | **<0.001** | 0.005 – 0.019 |
| Osbond acid (C22:5 n6) | 0.28 [0.046] | 0.34 [0.033] | **0.005** | 0.11 – 0.19 |
| Docosapentaenoic acid (DPA; C22:5 n3) | 1.05 [0.97,1.10] | 0.89 [0.80,0.98] | **0.018** | 0.90 – 1.56 |
| Docosahexaenoic acid (DHA; C22:6 n3) | 3.25 [0.37] | 1.86 [0.15] | **<0.001** | 1.08 – 2.16 |
| Lignoceric acid (C24:0) | 0.57 [0.50,0.68] | 0.26 [0.24,0.28] | **<0.001** | 0.22 – 0.37 |
| Nervonic acid (C24:1 n9) | 0.25 [0.031] | 0.19 [0.024] | **<0.001** | 0.24 – 0.35 |
|  |  |  |  |  |
| Total SFA | 51.7 [2.32] | 50.2 [1.75] | 0.13 | 46.8 – 51.3 |
| Total MUFA | 20.6 [1.12] | 24.7 [1.42] | **<0.001** | 15.2 – 16.4 |
| Total PUFA | 26.6 [1.67] | 24.3 [1.48] | **0.006** | 31.3 – 37.0 |
| Ratio ARA/DHA | 1.74 [0.18] | 2.51 [0.23] | **<0.001** | 4.11 – 5.48 |
| Ratio MA/ARA | 0.027 [0.005] | 0.025 [0.005] | 0.39 | 0.019 – 0.036 |
| Ratio n6/n3 | 3.55 [0.26] | 3.00 [0.37] | **0.002** | 6.52 – 8.77 |

Fatty acid composition (expressed as % total fatty acids) of phospholipids isolated from plasma samples. Data are expressed as mean [SD] or median [25th,75th percentile], depending on the underlying data distribution. P-values refer to the comparison SMOF vs. VEGA.

$, Values (range) from the reference sow-fed littermates (N=7).

Abbreviations: PN, parenteral nutrition; SMOF, piglets treated with SMOFlipid-based PN for 14 days; VEGA, piglets treated with Vegaven-based PN for 14 days.

**Table S7.** Fatty acid composition of liver triglycerides (% total fatty acids) on day 14

|  | **SMOF (N=9)** | **VEGA (N=10)** | **p-value** | **reference range$** |
| --- | --- | --- | --- | --- |
| Lauric acid (C12:0) | 0.130 [0.044] | 0.888 [0.379] | **<0.001** | 0.048 – 0.131 |
| Myristic acid (C14:0) | 2.34 [0.289] | 4.61 [0.799] | **<0.001** | 1.90 – 2.67 |
| Pentadecanoic acid (C15:0) | 0.16 [0.14,0.19] | 0.096 [0.08,0.12] | **0.003** | 0.131 – 0.144 |
| Palmitic acid (C16:0) | 20.3 [19.2,23.0] | 20.0 [18.9,24.7] | 0.94 | 30.8 – 34.8 |
| Palmitoleic acid (C16:1 n7) | 1.81 [0.24] | 1.48 [0.42] | 0.06 | 6.04 – 8.11 |
| Hypogeic acid (C16:1 n9) | 1.89 [0.24] | 2.06 [0.32] | 0.21 | 0.76 – 1.09 |
| Margaric acid (C17:0) | 0.25 [0.04] | 0.16 [0.04] | **<0.001** | 0.24 – 0.32 |
| Stearic acid (C18:0) | 5.70 [0.90] | 5.00 [1.59] | 0.27 | 5.86 – 8.38 |
| Oleic acid (C18:1 n9) | 23.0 [12.4,24.8] | 23.7 [21.8,25.9] | 0.51 | 22.8 – 25.2 |
| Vaccenic acid (C18:1 n7) | 2.23 [1.19,2.60] | 1.61 [1.33,2.05] | 0.14 | 2.96 – 3.38 |
| Linoleic acid (C18:2 n6) | 19.2 [1.70] | 10.2 [1.11] | **<0.001** | 10.9 – 13.2 |
| α-linolenic acid (ALA; C18:3 n3) | 1.62 [0.43] | 9.97 [3.92] | **<0.001** | 1.00 – 1.88 |
| γ-linolenic acid (GLA; C18:3 n6) | 0.59 [0.11] | 1.78 [0.40] | **<0.001** | 0.41 – 0.63 |
| Stearidonic acid (SDA; C18:4 n3) | 0.33 [0.31,0.41 | 1.55 [1.29,2.57] | **0.003** | 0.14 – 0.25 |
| Arachidic acid (C20:0) | 0.14 [0.033] | 0.12 [0.047] | 0.15 | 0.08 – 0.27 |
| Eicosadienoic acid (C20:2 n6) | 0.40 [0.11] | 0.13 [0.033] | **<0.001** | 0.17 – 0.30 |
| Dihomo-γ-linolenic acid (DGLA; C20:3 n6) | 0.95 [0.23] | 1.25 [0.26] | **0.015** | 0.18 – 0.34 |
| Mead acid (MA; C20:3 n9) | 0.078 [0.015] | 0.048 [0.010] | **<0.001** | 0.32 – 0.62 |
| Arachidonic acid (ARA, C20:4 n6) | 1.86 [1.60,1.98] | 1.72 [1.61,1.98] | 0.74 | 3.27 – 4.96 |
| Eicosatetraenoic acid (ETA; C20:4 n3) | 0.53 [0.11] | 4.57 [3.09,1.68] | **<0.001** | 0.03 – 0.12 |
| Eicosapentaenoic acid (EPA; C20:5 n3) | 1.55 [1.08,2.03] | 1.25 [1.10,1.59] | 0.68 | 0.16 – 0.37 |
| Adrenic acid (C22:4 n6) | 0.33 [0.04] | 0.049 [0.04] | **<0.001** | 0.019 – 0.03 |
| Osbond acid (C22:5 n6) | 0.22 [0.05] | 0.29 [0.15] | 0.21 | 0.11 – 0.17 |
| Docosapentaenoic acid (DPA; C22:5 n3) | 4.65 [1.16] | 3.24 [0.84] | **0.007** | 0.38 – 0.65 |
| Docosahexaenoic acid (DHA; C22:6 n3) | 6.98 [1.79] | 2.06 [0.96] | **<0.001** | 0.38 – 0.81 |
| Lignoceric acid (C24:0) | 0.112 [0.042] | 0.065 [0.036] | **0.019** | 0.09 – 0.12 |
| Nervonic acid (C24:1 n9) | 0.32 [0.11] | 0.15 [0.07] | **<0.001** | 0.26 – 0.35 |
|  |  |  |  |  |
| Total SFA | 31.1 [3.26] | 32.3 [3.86] | 0.20 | 40.0 – 45.5 |
| Total MUFA | 30.4 [2.13] | 29.2 [2.81] | 0.29 | 34.3 – 36.9 |
| Total PUFA | 39.8 [36.4,43.1] | 40.9 [32.9,43.8] | 1.00 | 18.1 – 22.8 |
| Ratio ARA/DHA | 0.25 [0.23,0.34] | 0.96 [0.79,1.25] | **<0.001** | 5.50 – 10.2 |
| Ratio MA/ARA | 0.043 [0.012] | 0.027 [0.005] | **0.001** | 0.08 – 0.15 |
| Ratio n6/n3 | 1.38 [1.30,1.82] | 0.70 [0.54,0.86] | **<0.001** | 4.62 – 7.46 |

Fatty acid composition (expressed as % total fatty acids) of triglycerides isolated from liver samples. Data are expressed as mean [SD] or median [25th,75th percentile], depending on the underlying data distribution. P-values refer to the comparison SMOF vs. VEGA.

$, Values (range) from the reference sow-fed littermates (N=7).

Abbreviations: PN, parenteral nutrition; SMOF, piglets treated with SMOFlipid-based PN for 14 days; VEGA, piglets treated with Vegaven-based PN for 14 days.

**Table S8.** Fatty acid composition of plasma triglycerides (% total fatty acids) on day 14

|  | SMOF (N=9) | VEGA (N=10) | p-value | reference range$ |
| --- | --- | --- | --- | --- |
| Capric acid (C10:0) | 6.72 [3.01] | 8.32 [2.90] | 0.25 | 5.27 – 11.9 |
| Lauric acid (C12:0) | 0.12 [0.07] | 0.26 [0.16] | 0.025 | 0.07 – 0.13 |
| Myristic acid (C14:0) | 1.57 [0.35] | 3.00 [0.83] | <0.001 | 2.03 – 2.77 |
| Pentadecanoic acid (C15:0) | 0.20 [0.047 | 0.13 [0.04] | 0.010 | 0.13 – 0.18 |
| Palmitic acid (C16:0) | 21.3 [3.88] | 22.4 [2.78] | 0.49 | 32.7 – 37.6 |
| Palmitoleic acid (C16:1 n7) | 1.94 [0.30] | 1.55 [0.45] | 0.044 | 7.42 – 9.38 |
| Hypogeic acid (C16:1 n9) | 1.17 [0.38] | 1.54 [0.21] | 0.015 | 0.56 – 0.77 |
| Margaric acid (C17:0) | 0.25 [0.21,0.28] | 0.18 [0.17,0.21] | 0.013 | 0.22 – 0.37 |
| Stearic acid (C18:0) | 7.41 [6.38,12.8] | 8.38 [5.43,10.3] | 0.94 | 5.09 – 9.73 |
| Oleic acid (C18:1 n9) | 24.3 [3.41] | 25.4 [3.25] | 0.49 | 23.6 – 27.8 |
| Vaccenic acid (C18:1 n7) | 1.80 [0.47] | 1.31 [0.35] | 0.023 | 2.48 – 2.83 |
| Linoleic acid (C18:2 n6) | 14.0 [12.4,14.3] | 8.48 [8.07,9.18] | <0.001 | 9.48 – 11.9 |
| α-linolenic acid (ALA; C18:3 n3) | 1.36 [0.36] | 9.39 [2.46] | <0.001 | 1.35 – 2.23 |
| γ-linolenic acid (GLA; C18:3 n6) | 0.43 [0.15] | 1.91 [0.36] | <0.001 | 0.26 – 0.49 |
| Stearidonic acid (SDA; C18:4 n3) | 0.51 [0.17] | 2.66 [0.77] | <0.001 | 0.11 – 0.19 |
| Arachidic acid (C20:0) | 0.21 [0.19,0.25] | 0.14 [0.12,0.16] | <0.001 | 0.10 – 0.16 |
| Eicosadienoic acid (C20:2 n6) | 0.15 [0.12,0.19] | 0.08 [0.06,0.12] | 0.003 | 0.13 – 0.24 |
| Dihomo-γ-linolenic acid (DGLA; C20:3 n6) | 0.47 [0.12] | 0.72 [0.09] | <0.001 | 0.12 – 0.24 |
| Mead acid (MA; C20:3 n9) | 0.089 [0.027] | 0.069 [0.033] | 0.15 | 0.22 – 0.72 |
| Arachidonic acid (ARA, C20:4 n6) | 1.53 [1.39,1.80] | 1.23 [0.96,2.14] | 0.10 | 1.26 – 2.36 |
| Eicosatetraenoic acid (ETA; C20:4 n3) | 0.34 [0.10] | 3.35 [1.11] | <0.001 | 0.023 – 0.059 |
| Eicosapentaenoic acid (EPA; C20:5 n3) | 8.32 [4.34] | 1.82 [0.62] | 0.002 | 0.16 – 0.54 |
| Adrenic acid (C22:4 n6) | 0.42 [0.074] | 0.067 [0.027] | <0.001 | 0.018 – 0.052 |
| Osbond acid (C22:5 n6) | 0.18 [0.057] | 0.21 [0.087] | 0.40 | 0.06 – 0.147 |
| Docosapentaenoic acid (DPA; C22:5 n3) | 2.17 [0.54] | 2.01 [0.59] | 0.55 | 0.21 – 0.38 |
| Docosahexaenoic acid (DHA; C22:6 n3) | 6.62 [1.50] | 1.98 [0.74] | <0.001 | 0.17 – 0.46 |
| Lignoceric acid (C24:0) | 0.14 [0.09,0.16] | 0.07 [0.06,0.12] | 0.05 | 0.05 – 0.09 |
| Nervonic acid (C24:1 n9) | 0.09 [0.08,0.12] | 0.08 [0.06,0.10] | 0.14 | 0.10 – 0.22 |
|  |  |  |  |  |
| Total SFA | 40.2 [10.0] | 42.9 [3.57] | 0.46 | 48.8 – 55.8 |
| Total MUFA | 29.6 [3.74] | 30.1 [3.28] | 0.78 | 36.9 – 40.1 |
| Total PUFA | 36.7 [32.4,42.5] | 37.7 [29.4,40.4] | 0.41 | 14.6 – 18.0 |
| Ratio ARA/DHA | 0.21 [0.15,0.28] | 0.78 [0.72,1.07] | 0.24 | 5.10 – 13.9 |
| Ratio MA/ARA | 0.056 [0.02] | 0.045 [0.02] | 0.001 | 0.17 – 0.33 |
| Ratio n6/n3 | 0.96 [0.27] | 0.68 [0.20] | 0.019 | 4.31 – 5.63 |

Fatty acid composition (expressed as % total fatty acids) of plasma triglycerides. Data are expressed as mean [SD] or median [25th,75th percentile], depending on the underlying data distribution. P-values refer to the comparison SMOF vs. VEGA.

$, Values (range) from the reference sow-fed littermates (N=7).

Abbreviations: PN, parenteral nutrition; SMOF, piglets treated with SMOFlipid-based PN for 14 days; VEGA, piglets treated with Vegaven-based PN for 14 days.

**Table S9.** Acycarnitines and other metabolites identified in brain tissue samples using the ionic metabolomic profiling approach (University of Alberta Metabolomics Centre/Nova Medical Testing Inc., Edmonton).

**Table S10.** Summary acylcarnitine data (computed from acylcarnitine data from Table S9).

|  | SMOF (N=9) | VEGA (N=10) | p-value | reference range$ |
| --- | --- | --- | --- | --- |
| free carnitine (C0)  (index of cellular fatty acid uptake) | 0.843 [0.136] | 0.841 [0.100] | 0.97 | 0.973 – 1.460 |
| total acylcarnitines (AC) | 0.376 [0.095] | 0.366 [0.054] | 0.78 | 0.319 – 0.619 |
| ratio AC/C0  (index of cellular fatty acid uptake) | 0.441 [0.060] | 0.435 [0.036] | 0.79 | 0.328 – 0.424 |
| total long chain acylcarnitines  (index of β-oxidation efficiency) | 0.100 [0.035] | 0.088 [0.015] | 0.37 | 0.076 – 0.143 |
| total very long chain acylcarnitines | 0.025 [0.006] | 0.023 [0.004] | 0.32 | 0.024 – 0.042 |
| total hydroxyacylcarnitines  (index of β-oxidation efficiency) | 0.064 [0.020] | 0.065 [0.016] | 0.92 | 0.056 – 0.145 |
| ratio C0/(C16 + C18)  (index of carnitine palmitoyltransferase I activity) | 11.4 [2.88] | 11.8 [1.23] | 0.68 | 12.3 – 18.2 |
| ratio (C16 +C18:1)/C2  (index of carnitine translocase activity) | 0.585 [0.176] | 0.460 [0.129] | 0.10 | 0.252 – 0.958 |
| C4-OH  (index of 3-hydroxyacyl-CoA dehydrogenase activity) | 0.055 [0.017] | 0.057 [0.014] | 0.81 | 0.050 – 0.131 |
| C6-OH  (index of 3-hydroxyacyl-CoA dehydrogenase activity) | 0.005 [0.002] | 0.004 [0.001] | 0.55 | 0.003 – 0.008 |

Data for single acylcarnitine species are provided as normalized intensity ratios, as in Table S9. Long chain acylcarnitines comprise species with 14 or more but less than 20 carbons. Very long chain acylcarnitines comprise species with 20 or more carbons. Data are expressed as mean [SD]. P-values refer to the comparison SMOF versus VEGA.

$, Values (range) from the reference sow-fed littermates (N=7).

Abbreviations: PN, parenteral nutrition; SMOF, piglets treated with SMOFlipid-based PN for 14 days; VEGA, piglets treated with Vegaven-based PN for 14 days.

**Table S11.** Metabolic hormones (insulin, glucagon, GLP-1)

|  | **SMOF (N=8)** | **VEGA (N=9)** | **p-value** |
| --- | --- | --- | --- |
| Insulin [mU/L] | 2.14 [0.43] | 2.07 [0.34] | 0.68 |
| #Glucagon [pg/mL] | 201.2 [80.5] | 126.8 [39.8] | **0.027** |
| #GLP-1 [pg/mL] | 59.7 [20.6] | 35.9 [23.4] | **0.042** |

Data are expressed as mean [SD]. #, One measurement in each group is missing due to sample degradation.

Abbreviations: GLP-1, glucagon-like peptide-1; PN, parenteral nutrition; SMOF, piglets treated with SMOFlipid-based PN for 14 days; VEGA, piglets treated with Vegaven-based PN for 14 days.

**
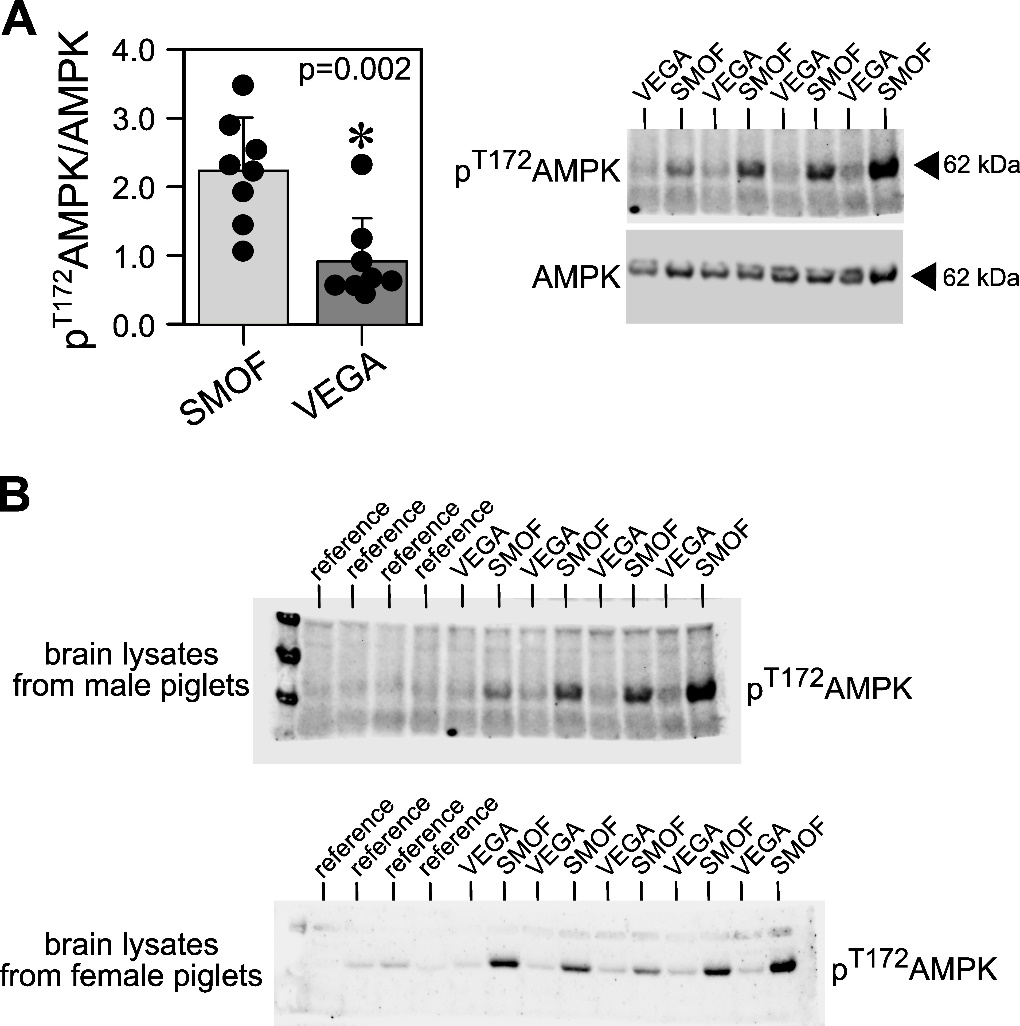
Figure S1.** AMP-activated protein kinase in brains of parenterally-fed piglets

(A) Activation of AMP-activated protein kinase (AMPK) in brain tissue lysates of male piglets treated with SMOFlipid-based PN (tissue samples from our previous study in male piglets see reference #11). *Significantly different. Bars represent means±SD. Dots indicate individual piglets. SMOF = male piglets treated with SMOFlipid-based parenteral nutrition (PN) for 14 days (N=8). VEGA = male piglets treated with Vegaven-based PN for 14 days (N=8). (B) Uncropped AMPK immunoblots of both male and female piglets including 4 reference samples from sow-fed littermates showing activation of AMPK brain samples in piglets treated with SMOFlipid-based PN.

**
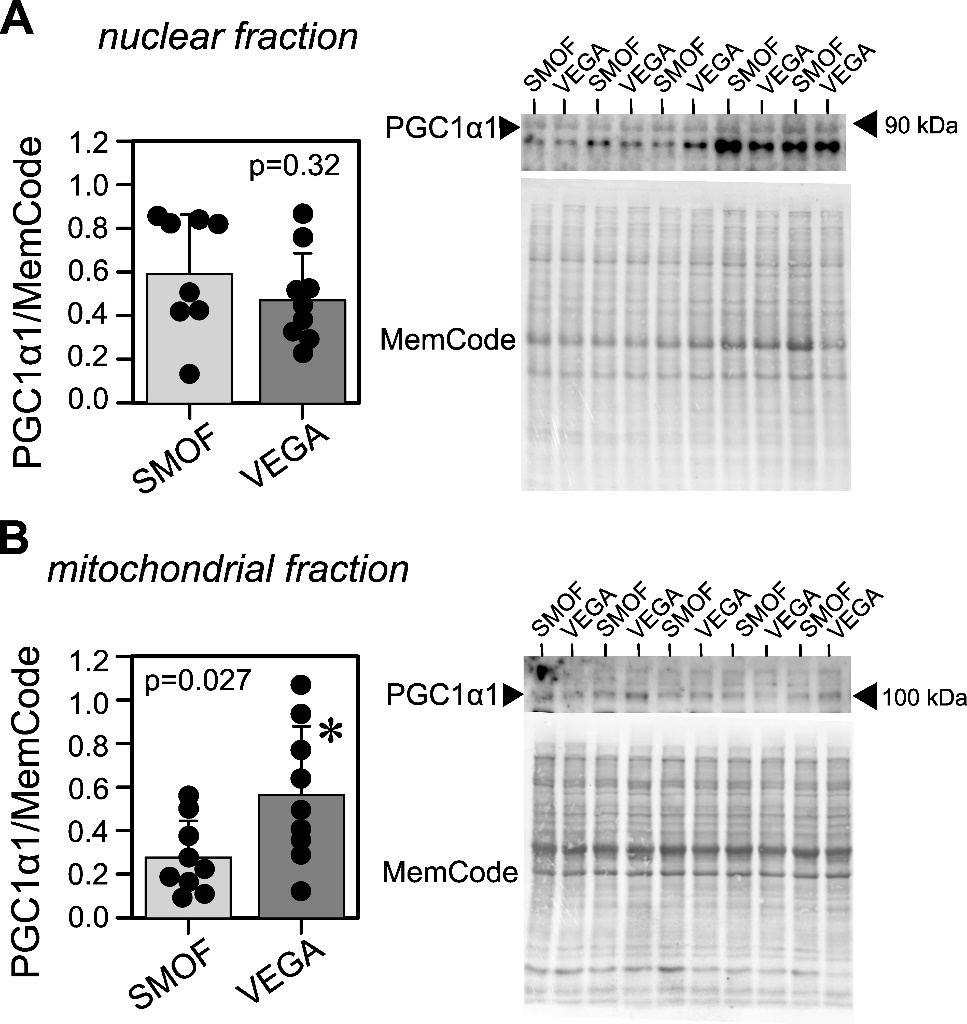
Figure S2.** Nuclear and mitochondrial translocation of PGC1α

Abundance of peroxisome proliferator-activated receptor gamma-coactivator 1 alpha (PGC-1α) in nuclear (A) and mitochondrial (B) fractions of brain tissue samples. *Significantly different. Bars represent mean±SD. Dots indicate individual piglets. SMOF = female piglets treated with SMOFlipid-based parenteral nutrition (PN) for 14 days. VEGA = female piglets treated with Vegaven-based PN for 14 days. N=9 for SMOF, N=10 for VEGA.

**
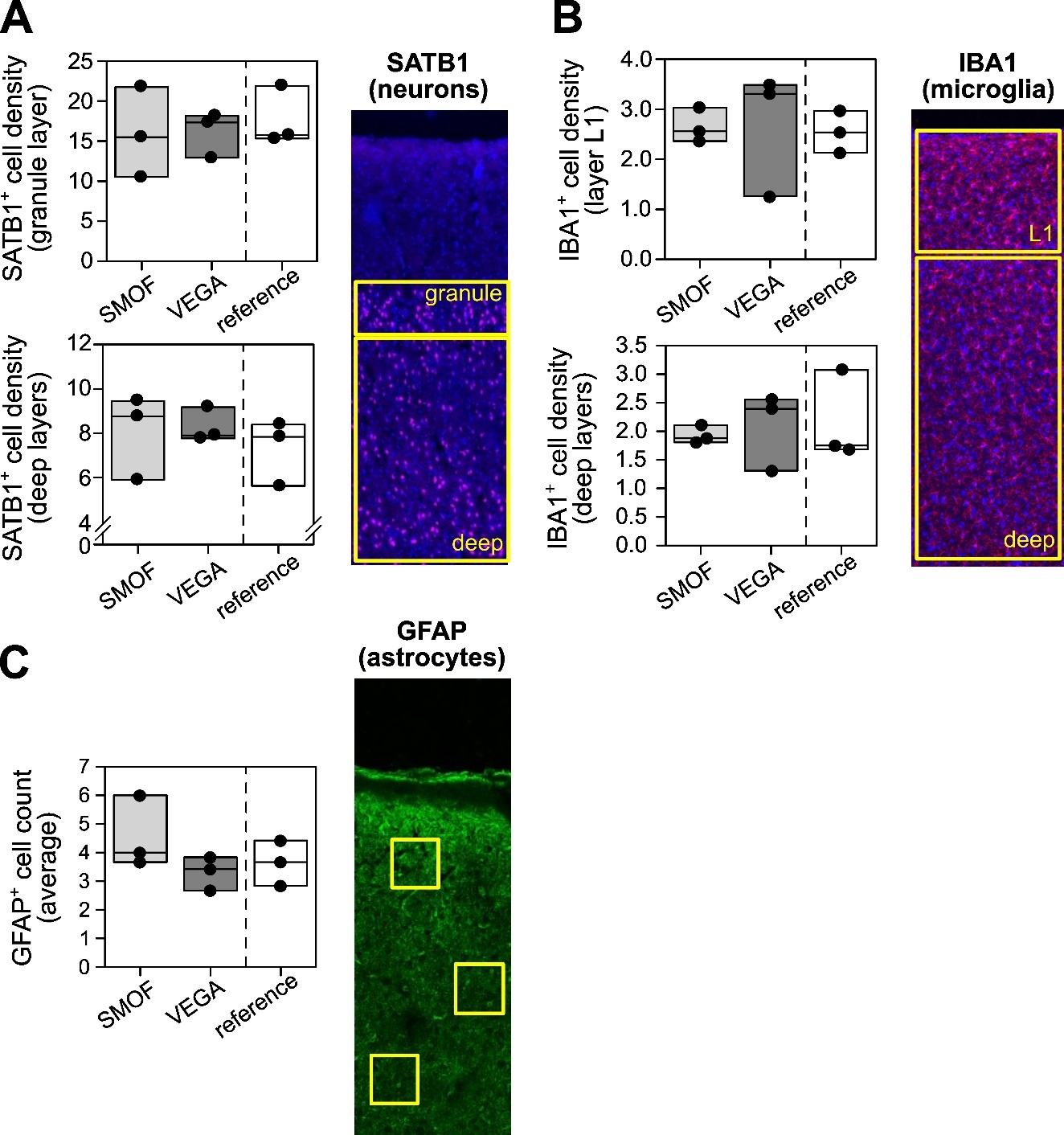
Figure S3.** Brain cell density studies

Immunohistochemical analysis of the frontal cortex. (A) Special AT-rich sequence binding protein 1 (SATB1) is expressed in excitatory neurons in the cortex. The number of positively stained cells per square millimeter was counted by using computer-assisted image analysis in the external granular layer of the cerebral cortex (granule) and the layers underneath (deep layers). (B) Ionized calcium-binding adapter molecule 1 (IBA1, microglia marker) staining. The number of positively stained cells per square millimeter was counted by using computer-assisted image analysis in the top layer of the cerebral cortex (L1) and the layers underneath (deep layers). (C) Glial fibrillary acidic protein (GFAP, astrocyte marker) staining. Data are expressed as the average of the number of GFAP-positive cells within each of 3 randomly selected frames (100µm x 100µm). Dots indicate individual piglets. SMOF, female piglets treated with SMOFlipid-based parenteral nutrition (PN) for 14 days (N=3); VEGA, female piglets treated with Vegaven-based PN for 14 days (N=3). Three age-matched sow-fed female piglets served as reference (control).


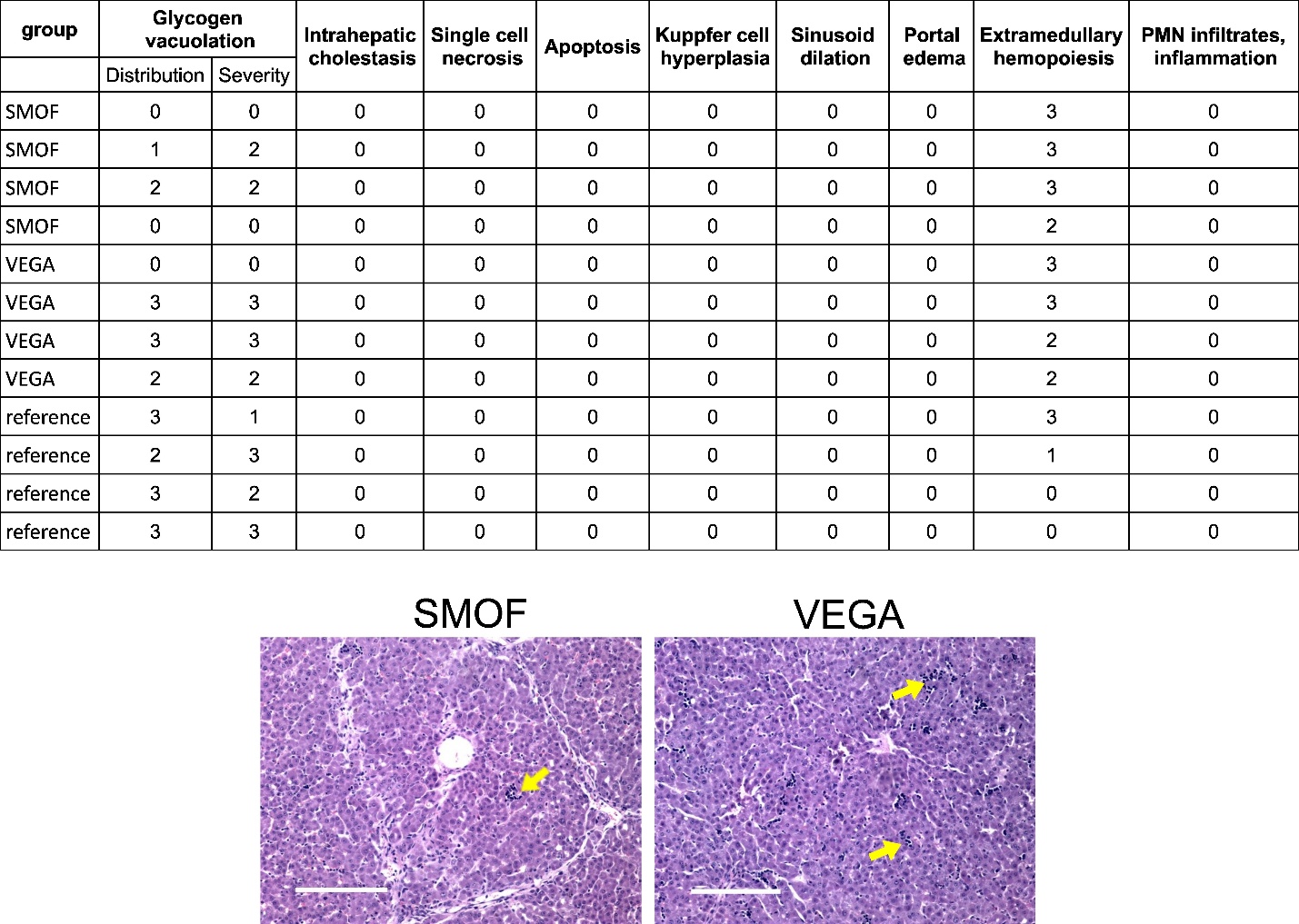
**Figure S4.** Liver histology

Liver histology results and representative sections (H.E. staining). Yellow arrows indicate clusters of extramedullary blood cell formation in neonatal livers. White bar in left lower corner indicates magnification (10 µm). Four age-matched sow-fed female piglets served as reference (control).

**Figure S5.** Liver RT-PCR results for IGF1, IGF1R, and IGFBP

**
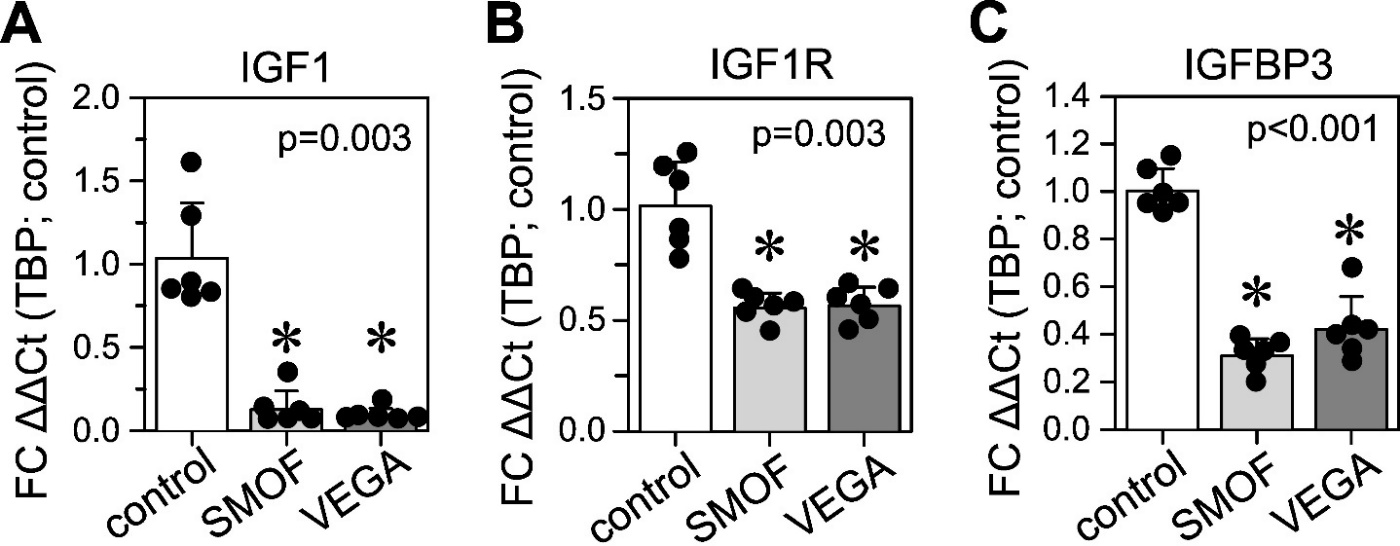
**

Quantification of transcripts for insulin like growth factor 1 (IGF1), insulin like growth factor 1 receptor (IGF1R), and insulin-like growth factor-binding protein 3 (IGFBP3) in liver tissue samples from randomly selected female piglets. SMOF, female piglets treated with SMOFlipid-based parenteral nutrition (PN) for 14 days (N=6); VEGA, female piglets treated with Vegaven-based PN for 14 days (N=6). Six age-matched sow-fed female piglets served as reference (control).


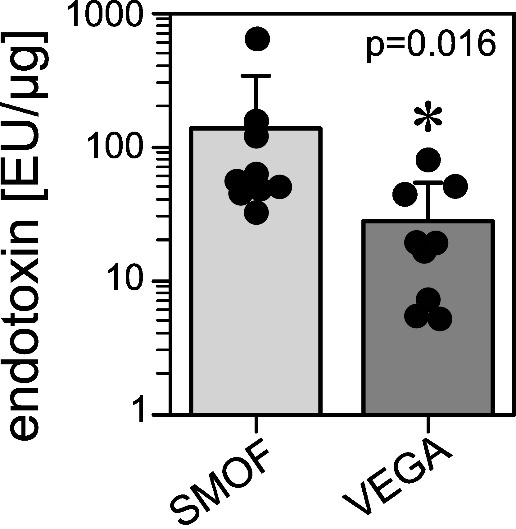
**Figure S6**. Pancreas endotoxin levels

LPS (endotoxin) tissue concentrations in total pancreatic tissue lysates. Note logarithmic scale of x-axis. *significantly different from SMOF. Bars represent means ± SDs. Dots indicate individual piglets. SMOF, female piglets treated with SMOFlipid-based PN for 14 days (N=9); VEGA, female piglets treated with Vegaven-based PN for 14 days (N=10).

**Figure S7.** Correlation between total bilirubin and glucagon plasma concentrations

**
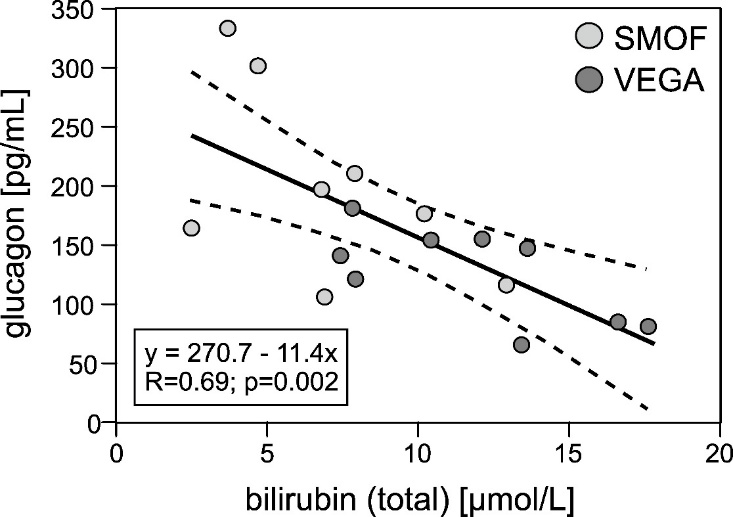
**

Correlation between total bilirubin and glucagon plasma concentrations in female piglets after parenteral nutrition (PN) for 14 days. The figure displays the scatter plot of corresponding measurements in individual piglets (dots) and their optimally fitted linear regression plot (line) with upper and lower 95% confidence intervals (dashed lines). Please note that 1 sample in each group is missing due to missing glucagon measurements because of sample degradation. SMOF, female piglets treated with SMOFlipid-based PN for 14 days (N=8); VEGA, female piglets treated with Vegaven-based PN for 14 days (N=9).
